# Cold‐pressed perilla seed oil: Investigating its protective influence on the gut–brain axis in mice with rotenone‐induced Parkinson's disease

**DOI:** 10.1002/fsn3.4265

**Published:** 2024-06-14

**Authors:** Peerapa Techaniyom, Chawin Korsirikoon, Thanaporn Rungruang, Narawut Pakaprot, Pinidphon Prombutara, Sujira Mukda, Aurawan Kringkasemsee Kettawan, Aikkarach Kettawan

**Affiliations:** ^1^ Doctor of Philosophy Program in Nutrition, Faculty of Medicine Ramathibodi Hospital and Institute of Nutrition Mahidol University Bangkok Thailand; ^2^ Department of Anatomy, Faculty of Medicine Siriraj Hospital Mahidol University Bangkok Thailand; ^3^ Department of Physiology, Faculty of Medicine Siriraj Hospital Mahidol University Bangkok Thailand; ^4^ OMICS Sciences and Bioinformatics Center, Faculty of Science Chulalongkorn University Bangkok Thailand; ^5^ Mod Gut Co., Ltd. Bangkok Thailand; ^6^ Research Center for Neuroscience Institute of Molecular Biosciences, Mahidol University Nakhon Pathom Thailand; ^7^ Institute of Nutrition, Mahidol University Nakhon Pathom Thailand

**Keywords:** alpha‐linolenic acid, disease, gut microbiome, gut–brain axis, nutrition, Omega‐3 PUFA, Parkinson's disease, Perilla seed oil, rotenone

## Abstract

Perilla seed oil, derived from a regional plant native to northern Thailand, undergoes cold‐pressing to analyze its bioactive components, notably alpha‐linolenic acid (ALA). ALA, constituting approximately 61% of the oil, serves as a precursor for therapeutic omega‐3 fatty acids, EPA and DHA, with neurodegenerative disease benefits and anti‐inflammatory responses. This study administered different concentrations of perilla seed oil to male C57BL/6 mice, categorized as low dose (LP 5% w/w), middle dose (MP 10% w/w), and high dose (HP 20% w/w), along with a fish oil (FP 10% w/w) diet. An experimental group received soybean oil (5% w/w). Over 42 days, these diets were administered while inducing Parkinson's disease (PD) with rotenone injections. Mice on a high perilla seed oil dose exhibited decreased Cox‐2 expression in the colon, suppressed Iba‐1 microglia activation, reduced alpha‐synuclein accumulation in the colon and hippocampus, prevented dopaminergic cell death in the substantia nigra, and improved motor and non‐motor symptoms. Mice on a middle dose showed maintenance of diverse gut microbiota, with an increased abundance of short‐chain fatty acid (SCFA)‐producing bacteria (*Bifidobacteria*, *Lactobacillus*, and *Faecalibacteria*). A reduction in bacteria correlated with PD (*Turicibacter*, *Ruminococcus*, and *Akkermansia*) was observed. Results suggest the potential therapeutic efficacy of high perilla seed oil doses in mitigating both intestinal and neurological aspects linked to the gut–brain axis in PD.

## Graphical abstract

Cold‐pressed perilla seed oil, rich in alpha‐linolenic acid, improved Parkinson's disease pathology and symptoms in rotenone‐induced mice via the gut–brain axis. It decreased alpha‐synuclein accumulation in the colon and hippocampus, reduced Cox‐2 expression, suppressed Iba‐1 microglia activation, prevented dopaminergic cell death in the substantia nigra, and improved neurobehavioral changes. Additionally, the oil maintained a diverse gut microbiota, promoting beneficial bacteria, and reducing PD‐associated bacteria (created by BioRender.com/Mahidol University).

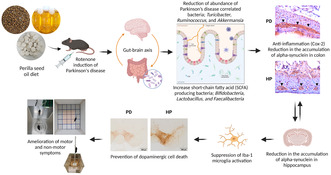

## 1 INTRODUCTION

Parkinson's disease (PD) is a progressive neurodegenerative movement disorder, with the incidence ranking second only to Alzheimer's disease. It predominantly affects the elderly, typically individuals aged 60–65 years and older, with a prevalence ranging from 1 to 3% in the population. Males exhibit 1–1.5 times higher susceptibility than females. The global estimate indicates that seven to ten million individuals are affected by PD (Kalia & Lang, 2015). The hallmark characteristic of PD involves the degeneration of dopaminergic neurons in the substantia nigra pars compacta (SNpc), responsible for dopamine production in the midbrain region of the brainstem. Additionally, the presence of α‐Synuclein (αSyn) inclusion bodies (Lewy pathology; LP) in neurons is a defining feature of PD. These factors lead to the death of dopaminergic cells and a subsequent reduction in dopamine levels. Dopamine plays a crucial role in various neurological processes, encompassing movement coordination, fine motor control, pleasure, cognition, memory, and learning. Despite extensive research, the etiology of PD remains elusive, and current therapeutic approaches only aim at impeding disease progression.

Furthermore, non‐motor symptoms are prevalent across all stages of PD, affecting individuals even in older age. In the early stages, manifestations may include gastrointestinal impairment, sleep disorders, hyposmia (a reduced sense of smell), depression, and anxiety. As the disease advances, classical motor symptoms such as resting tremor, muscle rigidity (stiffness), bradykinesia (slow movement), and postural instability (balance problems) become apparent. In later stages, individuals may experience mild cognitive impairment, known as Parkinson's disease dementia. Recognizing and addressing these non‐motor symptoms are pivotal for pre‐clinical diagnosis, preventive strategies, and improving the overall quality of life. Importantly, non‐motor symptoms can manifest several years before the onset of classical motor signs.

Research findings suggest that the etiological mechanisms implicated in PD encompass cellular oxidative stress, mitochondrial dysfunction, and neuronal inflammation. Reactive oxygen species generated during dopamine metabolism significantly contribute to the pathogenesis and synucleopathies associated with PD. Consequently, therapeutic strategies targeting the reduction of oxidative stress and augmentation of the antioxidant defense system have been proposed as potentially effective interventions for PD. Furthermore, a comprehensive investigation of alterations in gut microbiota composition, which instigate an imbalance of commensal gut microbes, increased intestinal permeability, and intestinal inflammation, has shed light on the connection between these factors and the accumulation of α‐synuclein in the intestinal and enteric nervous systems, contributing to the pathophysiology of PD (Kalia & Lang, 2015; Maiti et al., 2017; Poewe et al., 2017).

The genetic basis of Parkinson's disease (PD) is complex and multifaceted, with only a small percentage of cases attributed to autosomal dominant or recessive inheritance. Key genetic factors implicated in PD include chromosomal loci, referred to as genes located within the ‘PARK’ loci. Among these, monogenic forms of PD have been identified, including SNCA (α‐synuclein) and LRRK2 for autosomal dominant PD, and PINK1, PARK7 (DJ‐1), ATP13A2, and PARK2 (Parkin) for autosomal recessive PD. Notably, mutations in the SNCA gene, encoding α‐synuclein, were the first to be associated with inherited PD, with mutations in SNCA and LRRK2 commonly leading to Lewy pathology formation, characteristic of PD (Daher et al., 2012). Additionally, mutations in genes like PINK1, Parkin, and DJ‐1 are involved in oxidative stress mechanisms, suggesting a multifactorial interplay contributing to PD risk (Antony et al., 2013).

The transgenic A53T α‐synuclein mouse model is one of the most commonly used genetic models in the study of many aspects of PD. It can develop an overexpression of α‐synuclein and exhibit severe progressive motor impairment (Lee et al., 2002), mitochondrial alterations (Ordonez et al., 2018), neuroinflammation reactive gliosis, and impaired spatial memory (Zha et al., 2016). However, it is inconsistent to express the loss of dopaminergic cells in the substantia nigra (Javier Blesa & Przedborski, 2014). Therefore, the aim of our study is to focus on environmental factors of PD and the impact of diet on PD.

This study seeks to explore a neurotoxin‐based animal model that closely mimics the majority of hallmark pathologies observed in PD. The neurotoxin rotenone, employed to induce Parkinson's disease in mice, operates by selectively inhibiting complex I of the mitochondria in the substantia nigra (Xiong et al., 2012). This inhibition triggers a cascade of molecular pathological alterations, culminating in the degeneration of dopaminergic cells within the substantia nigra. (Blesa et al., 2012; Blesa & Przedborski, 2014; Gubellini & Kachidian, 2015; Jagmag et al., 2016; McDowell & Chesselet, 2012; Park et al., 2016; Ricardo et al., 2013; Xiong et al., 2012; Zeng et al., 2018).

This study aims to assess the neuroprotective effects of perilla seed oil, characterized by its high α‐linolenic acid (ALA) content, an n‐3 polyunsaturated fatty acid (PUFA). Functioning as an edible vegetable oil, perilla seed oil is considered an alternative to fish oil for therapeutic purposes. The evaluation will not only focus on motor symptoms but will also extend to non‐motor aspects, encompassing potential protective properties against inflammation, neuronal damage, α‐synuclein aggregation, and gut microbiota dysbiosis. Perilla, scientifically identified as Perilla frutescens or known as Nga‐mon in Thailand, belongs to the mint family, *Lamiaceae*, akin to basil. Its leaves and seeds have a longstanding tradition of use as an herbal remedy, with leaves consumed as an edible vegetable and seeds employed for cereal crops or oil extraction. Rich in bioactive substances, perilla includes phenolics and flavonoids like rosmarinic acid, luteolin, and apigenin. Remarkably, perilla seed oil stands out for having the highest concentration of omega‐3 PUFA, specifically α‐linolenic acid, among oilseed crops. Furthermore, the phytochemicals found in perilla have been associated with diverse bioactivities, including anti‐inflammatory, anti‐allergy, antioxidant (Kongkeaw et al., 2015), and anti‐cancer capacities (Pintha et al., 2018), demonstrated both in vitro and in vivo (Ahmed, 2018; Asif, 2011; Chumphukam et al., 2018; Igarashi & Miyazaki, 2013; Jayashree et al., 2014; Yu et al., 2017). Despite the abundance of beneficial biochemical compounds in perilla, there are limited studies exploring its physiological actions and developmental effects in animal studies, coupled with a lack of applications in clinical studies.

## 2 MATERIALS AND METHODS

### 2.1 Chemicals and reagents

Menhaden fish oil was procured from Sigma‐Aldrich (St. Louis, MO, USA), with product number F8020, CAS number 8002‐50‐4, and batch number SLCD8682. The oil comprises 25.9% omega‐3 fatty acids of the total fatty acid content. Soybean oil, containing 5% C18:3 linolenic acid as a source of omega‐3 fatty acids, was obtained from Thai Vegetable Oil PCL. (Bangkok, Thailand). Rotenone, Triton X‐100, and BSA were sourced from Sigma‐Aldrich Chemical Co. (St. Louis, MO, USA). Primary and secondary antibodies, along with chemical reagents for immunohistochemistry and Western blot analysis, were purchased from Vector Laboratories (Newark, CA, USA), Cell Signaling Technology (Danvers, MA, USA), and Bio‐Rad Laboratories (Hercules, CA, USA).

### 2.2 Plant oil material

The Thai perilla plant (*Perilla frutescens*) is cultivated and harvested in the local village of Pa Daet subdistrict, Mae Suai district, Chiang Rai province, Thailand, at the Agricultural Development Station situated on the heights of the royal initiative at Ban Huai Yuak Pa So. This cultivation takes place annually, from November to January. The harvested perilla plants are subsequently collected and subjected to drying. Voucher specimens of the perilla plant are stored at the Department of Pharmaceutical Botany, Faculty of Pharmacy, Mahidol University, with the reference number PBM no. 005745. The perilla seeds were dried prior to extraction using an expeller cold press machine at the Institute of Nutrition, Mahidol University, Salaya campus.

### 2.3 Animals and general procedures

Eight‐week‐old C57BL/6J male mice, weighing 23–25 g, were utilized in this study. A total of 90 mice were divided into 6 groups, with 15 mice per group. All mice were purchased from Nomura Siam International Co., Ltd., Bangkok, Thailand. The animals were housed in accordance with standard environmental conditions, including a room temperature and a 12‐h light–dark cycle, with free access to water and food. At the experiment's end, all mice were euthanized via an overdose of isoflurane inhalation. The mice were positioned supine and observed for any signs of awakening for a period of 3–5 min. The absence of a withdrawal reflex in their hind paws and urinary incontinence confirmed death. A midline incision was then made, and blood samples were collected through a cardiac puncture. Immediately after blood collection, caecum and colon samples were harvested. The mice were then perfused with 50 mL of ice‐cold 1X PBS to flush out the remaining blood in the body, followed by a 50 mL flush of 4% paraformaldehyde for fixation. The mice were then decapitated, and their perfused brains were collected. All experimental procedures involving animals were conducted with approval from the Siriraj Animal Care and Use Committee (Si‐ACUC), Faculty of Medicine, Siriraj Hospital, Mahidol University, Thailand (COA no. 006/2563).

### 2.4 Experimental design

**FIGURE 1:**
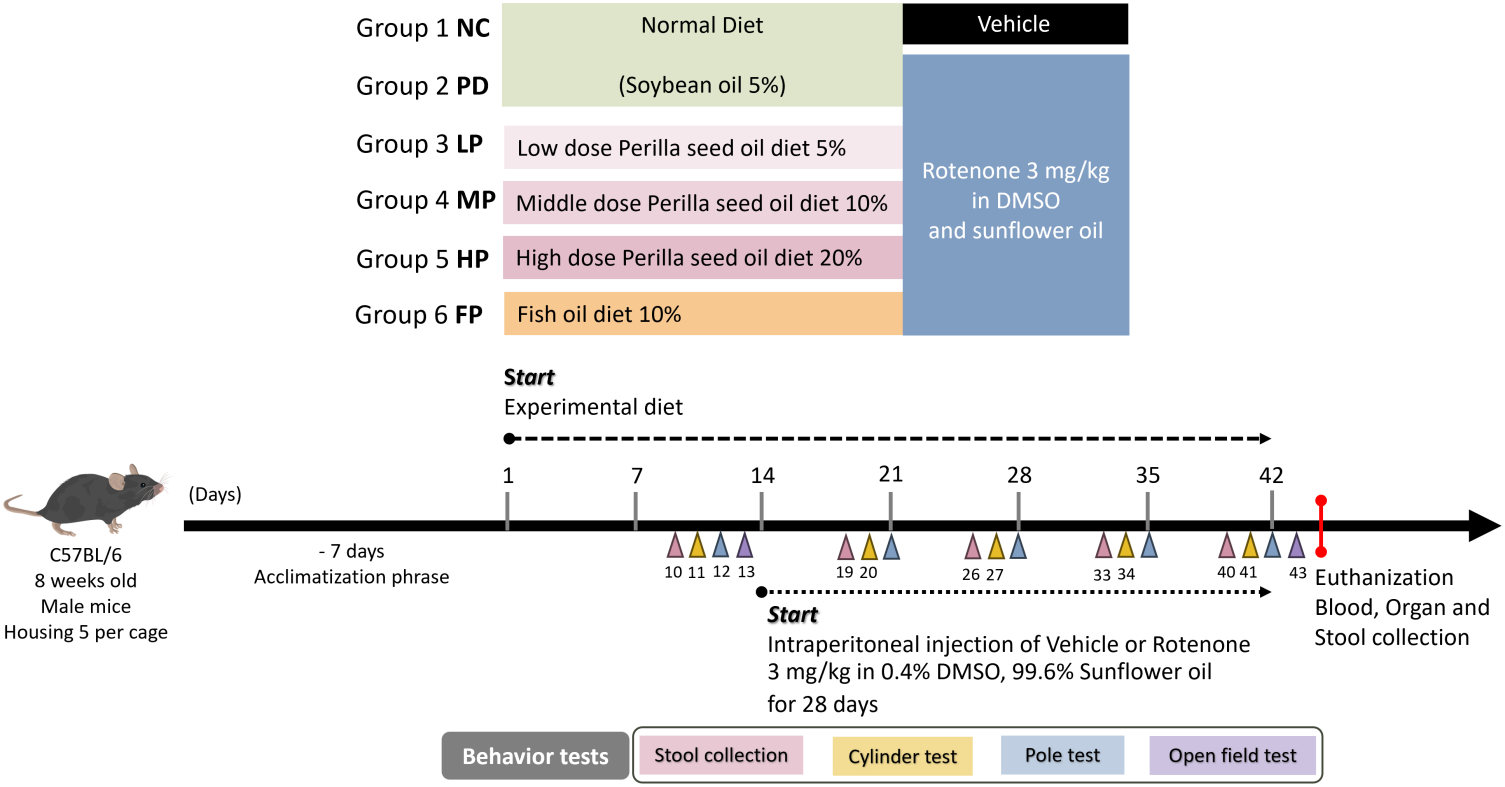
Overview of the study design.

After a 1‐week acclimatization period, the mice were placed on their respective experimental diets, comprising a control diet incorporating soybean oil, perilla oil, or fish oil. This dietary regimen was maintained for 2 weeks before the commencement of intraperitoneal injections of rotenone on day 15, a procedure that continued for the subsequent 28 days. This specified dietary condition was maintained throughout the 42‐day duration of the experiment. The group receiving an adequate intake of n‐3 PUFAs obtained perilla oil as a source of ALA and fish oil as sources of EPA and DHA. These edible oils replaced soybean oil, serving as both the total fat source and the source of n‐3 PUFAs in the standard AIN‐93 M diet (Figure 1).

### 2.5 Bioactive compounds of perilla oil measurement

#### 2.5.1 Fatty acid composition in perilla oil gas chromatography (GC) analysis

Combine 5 drops of perilla oil and 1 mL of KOH•MetOH in a glass tube. Mix the contents and heat the mixture in a water bath at 95–100°C for 5 min. Then, add 600 μL of BF_3_/MeOH reagent, mix again, and heat in the water bath for an additional 15 min. Afterward, add 1 mL of H_2_O to cool down the solution to room temperature, followed by extraction using 2.5 mL of petroleum ether. Mix immediately, collect the upper phase into a fresh tube, and incubate at 60°C overnight. To the resulting mixture, add 1 mL of internal standard C:17 (1 mg/mL), mix well, and transfer into a vial. Fatty acid methyl esters were analyzed by gas chromatography using a fully automated HP5890 system equipped with a flame‐ionization detector. Peaks were identified by comparing them with fatty acid standards (Nu‐chek‐Prep, Elysian, MN), and the area and percentage for each resolved peak were analyzed using a Perkin‐Elmer M1 integrator (Kang & Wang, 2005).

#### 2.5.2 Total tocopherol analysis

The total tocopherol content, encompassing α‐, β‐, γ‐, and δ‐tocopherol forms, in perilla oil was analyzed for vitamin E content using the high‐performance liquid chromatography (HPLC) method, modified from the procedure outlined by Fromm et al. (2012).

#### 2.5.3 Total phenolic acid and total flavonoid content analysis in perilla oil

The perilla seed oil samples were measured in triplicate for the determination of phenolic compounds, as modified from the previous method, using the Folin–Ciocalteu method (Medini et al., 2014). Additionally, total flavonoid analyses were conducted by the colorimetric method (Djeridane et al., 2006).

### 2.6 Antioxidant capacity analysis of perilla seed oil

#### 2.6.1 Oxygen radical absorbance capacity (ORAC) assay

The antioxidant capacity of perilla oil was determined using the oxygen radical absorbance capacity (ORAC) method, as established by Prior et al., 2003, and expressed in Trolox equivalents (μmol TE/g). The analysis was done in triplicate.

#### 2.6.2 Ferric reducing antioxidant power (FRAP) assay

Perilla oil was analyzed for its antioxidant capacity using the Ferric Reducing Antioxidant Power (FRAP) assay, adapted from the method described by Benzie and Strain (1996). FRAP values were determined via spectrophotometry, comparing the absorbance change at 593 nm in the test reaction mixtures with those containing known concentrations of ferrous ions.

### 2.7 Body weight and food intake

Food pellets were stored in mesh hoppers within each cage. Food intake was measured over a 24‐h period with mice housed in group cages, providing an average intake/mouse. Cumulative food intake was determined by subtracting the remaining food. Body weight was measured prior to the experiment and monitored daily (Yang et al., 2014).

### 2.8 Colon length

All mice were assessed for constipation symptoms, a prevalent digestive issue in non‐motor symptom PD patients. Subsequently, the colon length of mice was measured, and photographs were captured following their sacrifice. The colon was dissected from the ileocecum junction to the rectum (Perez‐Pardo, De Jong et al., 2017).

### 2.9 Neurobehavior test

#### 2.9.1 Pole test

The pole test is employed for the evaluation of bradykinesia and motor coordination in rodent models of Parkinson's disease, attributed to dopamine depletion. A metal pole, measuring 50 cm in length with a diameter of 10 mm, can be employed, designed with sufficient roughness to facilitate grip. During the pole test, the mouse is positioned on a vertical pole with its head facing downward, and the assessment is based on the time‐to‐reach‐the‐ground (T total). This time is measured with a maximum limit of 90 sec (Brooks & Dunnett, 2009; Ohno et al., 2008; Ruan & Yao, 2020; Strickland, 2014).

#### 2.9.2 Open‐field test

Mice will be placed within an apparatus comprising an arena enclosed by high walls to prevent escape. The floor of the open field is divided into inner and outer squares. This test employs a camera to quantify the movement of the test animal in both the peripheral and central zones of a 50 × 50 × 50 cm white polyvinyl chloride (PVC) box for a duration of 5 min. Changes in locomotion and other behaviors observed during this test can provide indications of neurological alterations (Barker et al., 2017; Gellért & Varga, 2016; Kraeuter et al., 2019; Przedborski & Vila, 2003; Seibenhener & Wooten, 2015; Valvassori et al., 2013).

#### 2.9.3 Cylinder test

The mouse is positioned within a glass cylinder (1‐liter glass beaker) in front of a mirror for 5 min. The frequency of times in which it rears up itself, with its paws making contact with the cylinder wall, is measured. Subsequently, an observer scores these wall touches by analyzing slow‐motion recorded videos (Magno et al., 2019; Smith & Heuer, 2011).

### 2.10 Immunohistochemistry analysis

The fixed tissues were immersed in 4% paraformaldehyde (4% PFA) and subsequently embedded in paraffin wax. Sections of 5 μm thickness were obtained using a microtome. The deparaffinization step involved immersion in xylene, followed by rehydration with alcohol. The samples on slides underwent antigen retrieval treatment with sodium‐citrate buffer at pH 6, heated at a temperature of 90–100°C for 5 min. Endogenous peroxidase activity was blocked by immersing the sections in 3% H_2_O_2_ in methanol at room temperature for 5 min. After washing with 1X PBS, membrane permeabilization was achieved by immersion in a solution of 1% Triton‐X100 in 1X PBS for 5 min. A non‐specific background was blocked using a BSA blocking solution at room temperature for 30 min. Primary antibody incubation was carried out overnight at 4°C in a moisture chamber. The primary antibodies used were rabbit anti‐tyrosine hydroxylase monoclonal antibody (#58844) at a 1:200 dilution and rabbit anti‐alpha synuclein monoclonal antibody (#4179) at a 1:500 dilution (Cell Signaling Technology). Following PBS washes, sections were incubated with a 1:500 dilution of goat anti‐rabbit IgG biotinylated secondary antibody for 2 h at room temperature. Signal amplification was achieved using the avidin–biotin conjugated to horseradish peroxidase method (Vectastain ABC Kit; Vector Laboratories Inc.). The presence of antibodies was detected by reaction with 3,3’‐diaminobenzidine (DAB substrate kit; Vector Laboratories Inc.; SK‐4100). Observation and image capture (.tiff file) were performed using the Olympus BX43 light microscope equipped with Olympus cellSens standard software. Data analysis was conducted using image J (NIH software) (Briscione et al., 2021; Schindelin et al., 2012; Seyed Jafari & Hunger, 2017) and the Qupath program (Bankhead et al., 2017; McCarty et al., 1985; McCarty & McCarty, 1984).

### 2.11 Western blot analysis

Animal tissues were lysed through ultrasonication in RIPA lysis buffer containing 1X protease inhibitor cocktail. The resulting tissue homogenate underwent centrifugation at 14,000 × *g* for 15 min at 4°C. Protein concentrations in the homogenized tissue were determined using the Bradford assay (Bio‐Rad, USA). The protein samples were mixed with Laemmli buffer and boiled at 95–100°C for 5 minutes before SDS‐PAGE electrophoresis. The transferred proteins were then transferred to a PVDF membrane using wash buffer (1X TBST). The target bands were incubated in blocking buffer (5% non‐fat dry milk in 1X TBST) at room temperature for 1 h. The membranes were washed twice for 5 min each in wash buffer (1X TBST). Following this, the membranes were incubated with specific target protein primary antibodies with the dilution 1:500 rabbit anti‐alpha synuclein monoclonal antibody (#4179), 1:1000 rabbit anti‐tyrosine hydroxylase monoclonal antibody (#58844), 1:1000 rabbit anti‐Cox‐2 monoclonal antibody (#12282), and 1:200 rabbit anti‐Iba1 monoclonal antibody (#17198) (Cell Signaling Technology). The primary antibodies were diluted in a solution containing 1% BSA in 0.1% Tween/TBS. After a further two washes with wash buffer (1X TBST) for 5 min each, the membranes were incubated with the secondary antibody anti‐rabbit IgG, conjugated with HRP (Cell Signaling Technology, #7074) at a dilution of 1:500 in 1X TBST. Subsequently, the membranes were washed twice for 5 min each in wash buffer (1X TBST). All immunoreactive bands were detected using the DAB substrate kit (Vector Laboratories) and peroxidase (HRP) (Vector Laboratories, SK‐4105) for 10 min, and the reaction was stopped with deionized water for 2 min. The membranes were scanned using an Epson scanner and printer (L3150) with a resolution of 2400 dpi, and the resulting .tiff files were analyzed for band densitometry and relative quantification using Image J software, with an internal standard protein.

### 2.12 Gut microbiome analysis

#### 2.12.1 Mice stool, cecal content, and DNA extraction

Stool samples from mice were collected from the caecum site after sacrifice, immediately kept at −80°C, and stored until analysis. The DNA will be extracted from the cecal content of mice. DNA extraction was performed using the DNA QIAamp PowerFecal Pro DNA Kit (Qiagen, Germany) following the manufacturer's instructions. The 16S ribosomal DNA (rDNA) sequences were amplified and pyrosequenced on an Illumina MiSeq platform. Pyrosequence reads were analyzed using Quantitative Insights into Microbial Ecology (QIIME) software version 22022.2. For taxonomic assignment, sequence reads were grouped into operational taxonomic units (OTUs) at a sequence similarity level of 97%. To assess the relative abundance of bacterial relationships between groups, 16S rRNA gene‐targeted RT‐PCR was performed. The copy number of target DNA was determined by comparing it with serially diluting standards (10^1^ to 10^7^ copies of plasmid DNA containing the respective amplicon for each set of primers). Bacterial quantity was expressed as Log_10_ (bacterial cells per gram of stool) (Tian et al., 2016).

#### 2.12.2 Bioinformatics analyses

The microbiome bioinformatics analysis was conducted using QIIME 22022.2 (Bolyen et al., 2019). Initial processing involved demultiplexing raw sequence data through the q2‐demux plugin, and reads with expected errors (maxEE) higher than 3.0 were eliminated by the denoising software, DADA2 (via q2‐dada2). Additionally, 16S sequences associated with chloroplasts were removed. The construction of a phylogeny from representative sequences was accomplished using the align_to_tree_mafft_fasttree action within the q2‐phylogeny plugin. For diversity analysis, alpha‐diversity metrics, beta‐diversity metrics, and principal coordinate analysis (PCoA) were estimated using the q2‐diversity tool after rarefying (subsampling without replacement) the samples to 32,366 reads. Taxonomic assignment to ASVs was performed using the classify‐sklearn naive Bayes taxonomy classifier against the Greengenes 13_8 99% OTU reference sequences. Statistical tests of alpha and beta diversity were performed using Kruskal–Wallis and PERMANNOVA (number of permutations = 999), respectively. Moreover, the significantly differential abundance analysis of microbiota was carried out using linear discriminant analysis effect size (LEfSe) (Segata et al., 2011). First, nonparametric factorial Kruskal−Wallis sum‐rank tests were employed to choose features differentially distributed among classes (*p* < .05). Subsequently, a linear discriminant analysis (LDA) model estimated their effect sizes, supported by 30‐fold bootstrapping (cutoff = logarithmic LDA score of ≥2.0). In addition, pairwise heat tree analysis was performed, leveraging the hierarchical structure of taxonomic classifications to quantitatively (using the median abundance) and statistically (using the non‐parametric Wilcoxon Rank Sum test) depict taxonomic differences between microbial communities for the experimental groups (Foster et al., 2017).

### 2.13 Data analysis and statistical methods

The results are expressed as mean ± S.D. or mean ± SEM, as indicated. Group differences were assessed through a one‐way analysis of variance (ANOVA), followed by post‐hoc Tukey multiple comparison tests or Dunnett T3 correction for multiple comparisons in cases where the data demonstrated a normal distribution. For non‐normally distributed data, the Kruskal–Wallis test was employed, followed by Dunn's correction for multiple comparisons. Statistical significance was defined at p‐values <0.05. All statistical analyses were performed using the GraphPad Prism program, version 10.1.2 (324).

## 3 RESULTS

### 3.1 Fatty acid composition in cold‐pressed perilla seed oil

Table 1 presents the quantitative results of 6 measured fatty acids. The major fatty acids identified in perilla seed oil through gas chromatography include linolenic acid (61.36 ± 0.42%), linoleic acid (19.66 ± 0.07%), and oleic acid (9.52 ± 0.10%), respectively.

**TABLE 1:** Fatty acid contents of perilla seed oil.

Common name	Fatty acid symbol	Fatty acid content^a^ (%)
Octanoic (caprylic)	C8:0	1.07 ± 0.02
Hexadecanoic (palmitic)	C16:0	6.94 ± 0.10
Octadecanoic (stearic)	C18:0	2.52 ± 0.30
Cis‐9‐Octadecenoic (oleic)	C18:1 n9 cis	9.52 ± 0.13
Cis‐9,12‐Octadecadienoic (linoleic)	C18:2 n6 (c9 c12)	18.59 ± 0.17
Cis‐9,12,15‐Octadecatrienoic (linolenic)	C18:3 n3	61.36 ± 0.42

^a^ Values are the means ± SD of three (*n* = 3) measurements.

### 3.2 Quantitative composition of total tocopherol, total phenolic compound, and total flavonoid content of perilla seed oil

The total tocopherol, total phenolic compound, and total flavonoid contents of perilla seed oil are summarized in Table 2. The analysis of perilla seed oil revealed a total tocopherol content of 783.90 ± 10.35 mg/kg of oil, a total phenolic compound content of 23.46 ± 2.62 mg GAE/g, and a total flavonoid content of 0.11 ± 0.02 mg RE/g.

**TABLE 2:** Contents of bioactive compounds in perilla seed oil (mg/kg oil).

Bioactive compound	Quantitative^a^
Total tocopherol (mg/kg)	783.90 ± 10.35
Total phenolic compound (mg GAE/g)	23.46 ± 2.62
Total flavonoid content (mg RE/g)	0.11 ± 0.02

^a^ Values are the means ± SD of three (*n* = 3) measurements.

### 3.3 Antioxidant capacity of perilla seed oil

The two different methods of antioxidant capacity analysis are shown in Table 3. The antioxidant capacity using the ORAC assay is 161.07 ± 6.94 μmol TE/g and the FRAP assay is 7.57 ± 0.08 μmol TE/g.

**TABLE 3:** Antioxidant capacity of perilla seed oil.

Method	Antioxidant activity^a^
Oxygen Radical Absorbance Capacity (ORAC) (umol TE/g)	161.07 ± 6.94
Ferric reducing antioxidant power (FRAP) (umol TE/g)	7.57 ± 0.08

^a^ Values are the means ± SD of three (*n* = 3) measurements.

### 3.4 Change in animal body weight and food intake

**FIGURE 2:**
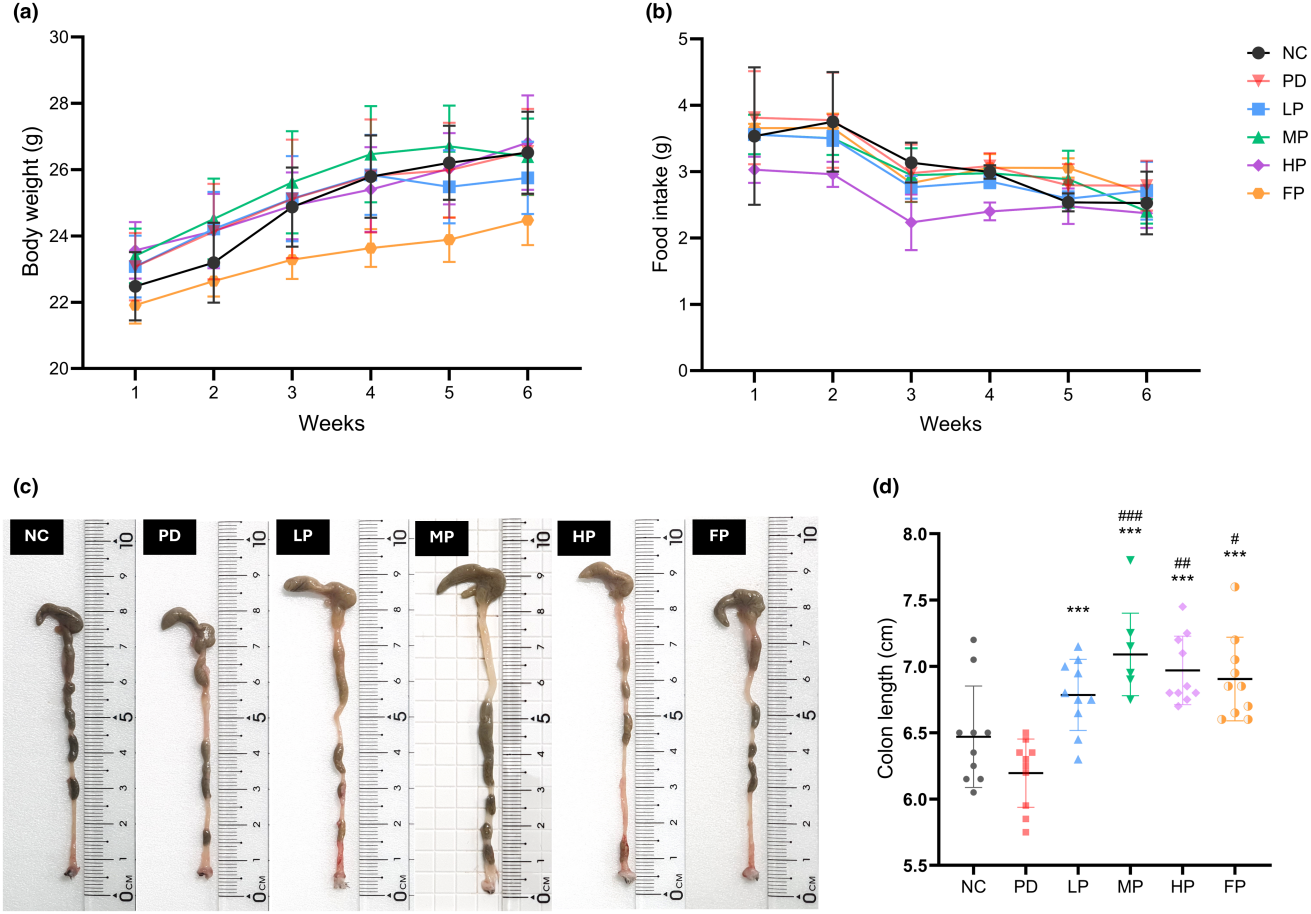
Growth chart reflecting actual body weight (a) and food intake per mouse (b) in each group, observed weekly. The data for each timepoint were expressed as mean ± SD, with a sample size of 10 for the NC group and 14–15 for the PD, LP, MP, HP, and FP groups. Fresh mouse colon samples were harvested during necropsy (c), and a dot‐plot represents the effect of edible oil‐formulated diets on average colon length (d) in a mouse model of rotenone‐induced Parkinson's disease (*n* = 10). The data were analyzed by one‐way ANOVA with Tukey multiple comparisons and are expressed as mean ± SD. Statistical significance is indicated by *p < .05, **p < .01, ***p < .001 vs PD group; ^#^ *p* < .05, ^##^ *p* < .01, ^###^ *p* < .001 vs NC group.

The administration of rotenone for the induction of Parkinson's disease did not exert discernible effects on alterations in the body weight of the experimental animals. Over the course of their growth, the body weight of all mice exhibited an increase, with the exception of those assigned to the fish oil group. This discrepancy in weight dynamics suggests a potential influence of divergent dietary oil compositions on the observed variations in body weight. Notably, the body weight of the FP group remained significantly lower throughout the study duration. At the 6‐week juncture, the body weight gain of the FP group exhibited statistical significance in contrast to each of the comparator groups: NC 26.51 ± 1.24 g vs. FP 24.48 ± 0.75 g, **p = .04; PD 26.55 ± 1.29 g vs. FP 24.48 ± 0.75 g, ***p < .001; LP 25.75 ± 1.09 g vs. FP 24.48 ± 0.75 g, *p = .013; MP 26.39 ± 1.15 g vs. FP 24.48 ± 0.75 g ***p < .001; HP 26.82 ± 1.43 g vs. FP 24.48 ± 0.75 g, ***p < .001 (Figure 2a). All mice showed an increase in body weight over time (Figure 2a). However, the MP group had the lowest average body weight gain (3.06 ± 0.98 g), significantly lower than the NC (4.60 ± 1.07 g, *p* = .021) and PD (4.65 ± 1.12 g, *p* = .005) groups. The FP group also had a significantly lower body weight gain (3.21 ± 0.97 g) compared to the PD group (4.65 ± 1.12 g, *p* = .016) (Figure S1a).

The outcomes pertaining to body weight and dietary intake manifest a discernible correlation, as illustrated in Figure 2a,b. Weekly food intake analysis (Figure 2b) revealed that the HP group exhibited lower food consumption compared to all other groups, with significant differences observed at week 4 (PD: 3.09 ± 0.19 g vs. HP: 2.10 ± 0.13 g, *p < .05; MP: 2.98 ± 0.08 g vs. HP: 2.10 ± 0.13 g, *p = .031). The HP group demonstrated significantly lower average food consumption compared to all other groups (*p* < .001), as depicted in Figure S1b. However, no significant differences were found in the average energy intake per mouse (Figure S1c). Despite this, the HP group had the highest average energy intake from fat (4.472 ± 0.72 kcal/mouse), significantly more than all other groups (*p* < .001). The MP and FP groups had similar fat energy intakes (2.742 ± 0.41 kcal/mouse and 2.732 ± 0.51 kcal/mouse, respectively), which were significantly higher than the NC, PD, and LP groups (*p* < .001). There were no significant differences in fat energy intake between the NC, PD, and LP groups. This pattern was also observed in the average fat consumption per mouse. Regarding n‐3 PUFA intake, the LP group consumed significantly more than the NC and PD groups (*p* < .001), even with similar fat intakes. The LP group also consumed more n‐3 PUFA than the FP group (*p* < .001), despite consuming less fat. The MP and HP groups consumed significantly more PUFA than the FP group (*p* < .001 for both comparisons), with the HP group consuming the most PUFA of all groups (*p* < .001). These findings are summarized in Table S2.

### 3.5 Effect of perilla seed oil and fish oil diets on increasing colon length in mice with rotenone‐induced Parkinson's disease

To elucidate the influence of intraperitoneal rotenone administration on constipation symptoms, a prevalent manifestation in PD patients (Pedrosa Carrasco et al., 2018), we explored the correlation between PD and dietary treatment with intestinal motility by observing changes in colon length (Figure 2c,d). The outcomes revealed a noteworthy increase in colon length within the MP group as compared to the NC and PD groups (NC 6.47 ± 0.38 cm vs. MP 7.09 ± 0.31 cm, ^###^ *p* < .001, PD 6.20 ± 0.26 cm vs. MP 7.09 ± 0.31 cm, ***p < .001). Correspondingly, the HP and FP groups exhibited a significant elongation in colon length relative to the NC and PD groups (NC 6.47 ± 0.38 cm vs. HP 6.97 ± 0.26 cm, ^##^ *p* = .006, PD 6.20 ± 0.26 cm vs. HP 6.97 ± 0.26 cm, ***p < .001, NC 6.47 ± 0.38 cm vs. FP 6.91 ± 0.31 cm, ^#^P = .025, PD 6.20 ± 0.26 cm vs. FP 6.91 ± 0.31 cm, ***p < .001).

### 3.6 Perilla oil‐based diet mitigated behavioral impairments caused by rotenone induction

#### 3.6.1 Pole test

**FIGURE 3:**
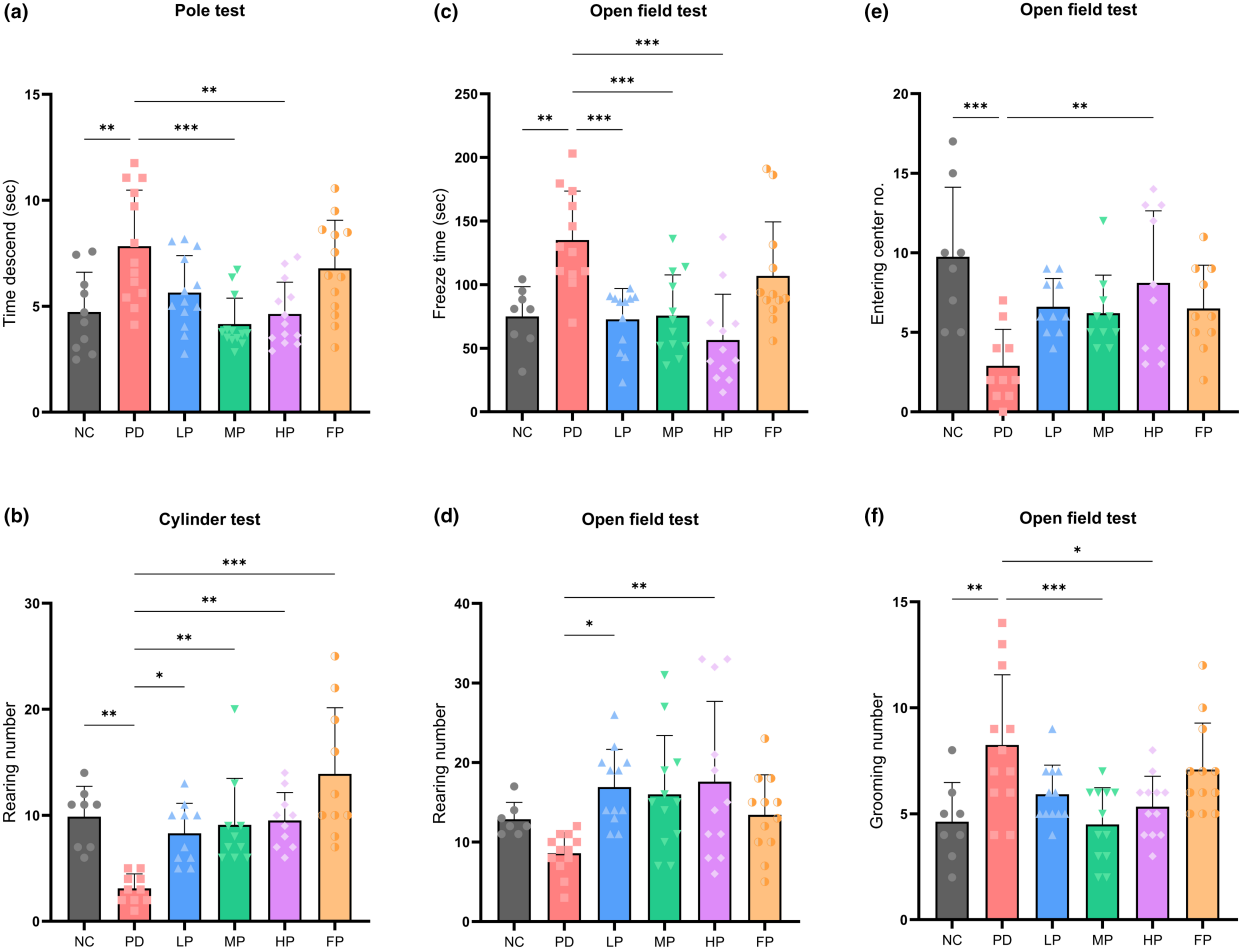
Effects of diets formulated with various edible oils on rotenone‐induced motor impairment and neurobehavioral changes in mice. The descending time mice spent on the pole test is shown in (a), with a sample size of 10 for NC and 13 for PD, LP, MP, HP, and FP. The cylinder test, used to assess spontaneous exploratory behavior, counted the number of rearing instances from both forelimbs of each mouse that touched the cylinder wall (b); NC, *n* = 8, PD, LP, MP, HP, and FP, *n* = 10. The open field test demonstrated mouse freezing time (c), number of rearing instances touching the arena wall (d), number of entries to the central zone (e), and grooming frequencies (f) for each study group (*n* = 7–12). The data, analyzed via one‐way ANOVA followed by Tukey's multiple comparisons, is expressed as mean ± SD. Asterisks indicate significant differences (*p < .05, **p < .01, ***p < .001).

The pole test was employed for the assessment of motor impairments, specifically bradykinesia (characterized by slow movement), in mice afflicted with rotenone‐induced Parkinson's disease (Figure 3a). In the final week of testing, Dunnett's multiple comparison test revealed a noteworthy deceleration in movement within the PD group. Mice within this group exhibited a mean descent time of 7.831 ± 2.647 s to descend the pole, compared to the NC group, which demonstrated a mean descent time of 4.727 ± 1.873 s (**p < .001). Furthermore, the PD group displayed significant differences in descent time when compared to the MP group (7.831 ± 2.647 s vs. 4.157 ± 1.225 s, ***p < .001), as well as the HP group (7.831 ± 2.647 s vs. 4.633 ± 1.503 s, **p = .001).

#### 3.6.2 Cylinder test

The cylinder test was utilized to evaluate the exploratory behavior of mice. As depicted in Figure 3b, upon completion of the experiment, the PD group manifested a statistically significant reduction in the frequency of rearing in comparison to all other experimental groups. Specifically, the PD group exhibited a mere 3.100 ± 1.370 rearing, while the NC, LP, MP, HP, and FP groups demonstrated 9.875 ± 2.850, 8.300 ± 2.830, 9.100 ± 4.383, 9.500 ± 2.635, and 13.90 ± 6.244 rearing, respectively (**p = .005, *p = .035, **p = .009, **p = .005, ***p = .001, respectively).

#### 3.6.3 Open field test

The open‐field test was employed to evaluate spontaneous exploration and emotional responses within distinct experimental groups. Analysis of freezing times revealed that each group exhibited an increased duration of rest, freezing, or stopping of locomotion within the peripheral area of the testing arena. Notably, the PD group demonstrated significantly prolonged freezing times compared to the other groups (Figure 3c). The PD group displayed a mean freezing time of 135.0 ± 38.51 s, while the NC, LP, MP, and HP groups showed mean freeze times of 74.93 ± 23.51, 72.76 ± 24.14, 75.58 ± 32.12, and 56.49 ± 35.95 s, respectively (*p = .04, ***p < .001, ***p < .001, and ***p < .001, respectively). The experimental groups consisted of NC (*n* = 8), PD, LP, MP, HP, and FP (*n* = 12).

The open field test facilitates the assessment of rearing frequency, as illustrated in Figure 3d. The rearing activity of the PD group correlates with the freeze time data, indicating that an increased duration of stopping or resting among PD mice is associated with a reduction in rearing behavior. In addition, the PD group showed a statistically significant decrease in rearing frequency compared to the LP and HP groups. The rearing numbers were 8.583 ± 2.61 for the PD group, 16.92 ± 4.783 for the LP group (*p = 02), and 17.58 ± 10.09 for the HP group (**p = 0.009). Notably, the differences in rearing frequency between PD and LP, as well as PD and HP groups, are statistically significant. The experimental groups included normal control (NC, *n* = 7) and PD, LP, MP, HP, and FP groups (*n* = 12 each).

In the open‐field test, mice exhibit an inclination to traverse the outer periphery of the testing arena as opposed to the central region. Those demonstrating anxiety‐like behavioral traits manifest reduced exploration within the central area of the arena, an increased thigmotaxis (i.e., preference for the periphery), and decreased rearing behavior. As delineated in Figure 3e, the NC group exhibited a markedly elevated frequency of entries into the central zone: NC 9.750 ± 4.367 as compared to PD 2.900 ± 2.283, with a statistical significance of ***p = .001; further, PD 2.900 ± 2.283 vs. HP 8.100 ± 4.533 demonstrated statistical significance at **p = .006. The group sizes for NC and experimental groups (PD, LP, MP, HP, and FP) were *n* = 8 and *n* = 10, respectively.

The analysis of grooming numbers serves as an indicator of anxiety‐like behavior or emotional response within a group of mice exposed to a controlled environment. The graphical representation in Figure 3f illustrates a statistically significant increase in grooming numbers within the PD group in comparison to the NC, MP, and HP groups. Specifically, the grooming numbers were observed as PD 8.250 ± 3.306 vs. NC 4.625 ± 1.847 **p = .005, PD 8.250 ± 3.306 vs. MP 4.500 ± 1.732 ***p < .001, and PD 8.250 ± 3.306 vs. HP 5.333 ± 1.435 *p = .014. The sample sizes for NC and experimental groups (PD, LP, MP, HP, and FP) were *n* = 8 and *n* = 12, respectively.

### 3.7 Immunohistochemical analysis of brain and colon tissues in a Parkinson's disease mouse model

#### 3.7.1 Tyrosine hydroxylase loss in the nigrostriatal pathway

**FIGURE 4:**
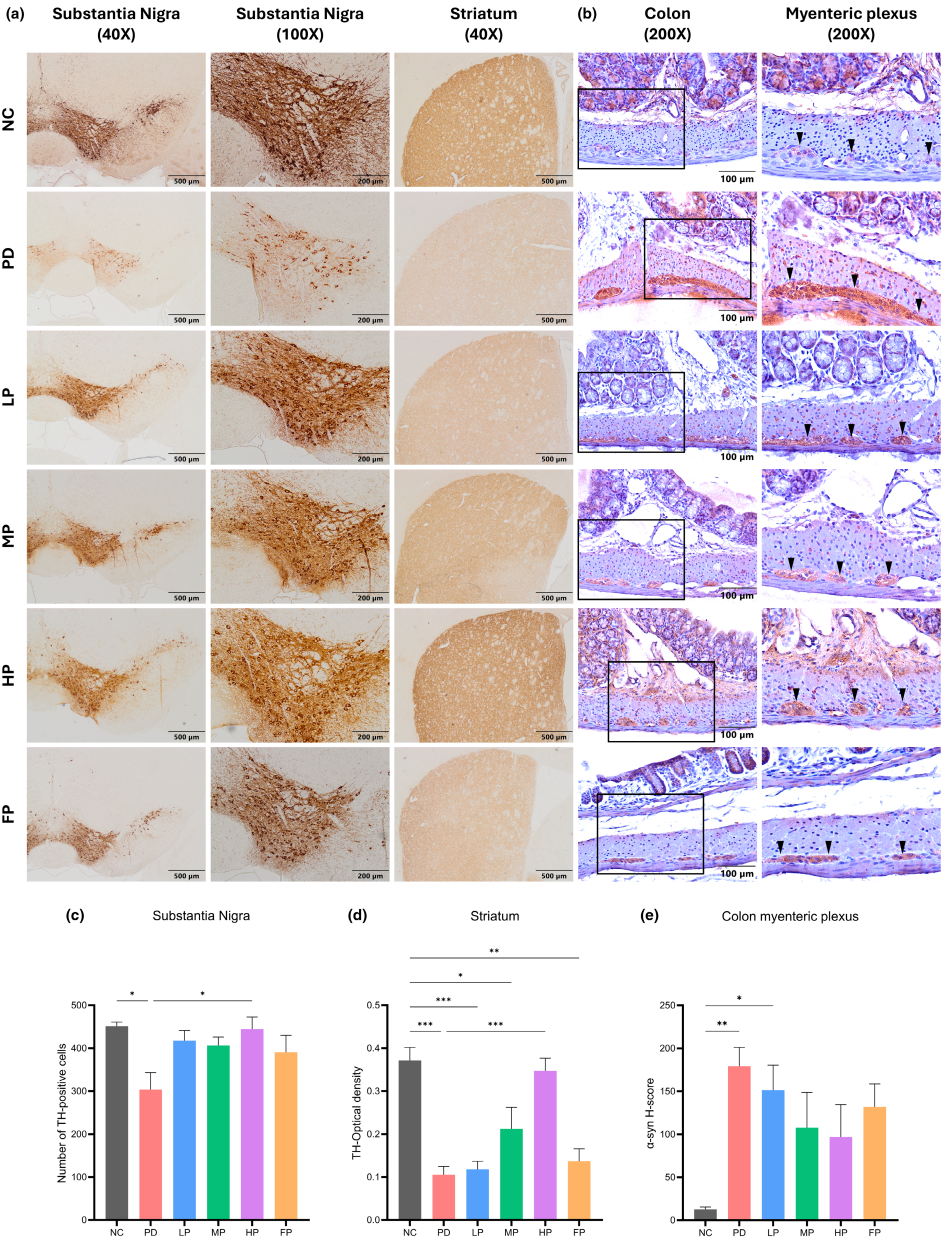
Photomicrographs show immunohistochemistry staining for tyrosine hydroxylase (TH) in mouse substantia nigra and striatum sections (a) and α‐synuclein in mouse colon sections (b). Mouse brain samples, sectioned in a coronal plane, exhibited a dark brown color, indicating the presence of TH‐positive neurons and their fibers. Colon samples were sectioned in a longitudinal plane and counterstained with Carazzi's hematoxylin. Black arrows indicate the myenteric (Auerbach) plexus ganglia located between the inner circular and outer longitudinal smooth muscle fibers of the intestinal muscularis propria. Scale bars of 500 μm, 200 μm, and 100 μm indicate magnifications of 40X, 100X, and 200X under the light microscope, respectively. Bar plots show TH‐positive cells in the substantia nigra (c), TH optical density in the striatum (d), and the H‐score of α‐synuclein immunoreactivity in the mouse colon myenteric plexus (e). The data were analyzed using one‐way ANOVA with Tukey multiple comparisons and are expressed as mean ± SEM (*n* = 5 for substantia nigra, *n* = 3–5 for striatum, and *n* = 4–5 for colon). Asterisks indicate significant differences (*p < .05, **p < .01, ***p < .001).

Figure 4a exhibits images of immunoreactivity expression in the substantia nigra, showcasing TH‐positive cells at magnifications of 40× and 100×, respectively. Dopaminergic neurons, characterized as multipolar neurons, displayed distinctive features with a large, round cell body containing a single nucleus and several branching dendrites. Notably, these neurons exhibited a distinctive cytoplasm positively stained for tyrosine hydroxylase (TH), the rate‐limiting enzyme in dopamine synthesis. The findings unveiled a substantial degeneration of dopaminergic cells following 4 weeks of exposure to rotenone. TH immunoreactivity served as a reliable marker for the identification of dopaminergic neurons and their axonal projections within the brain. As illustrated in Figure 4c, quantification of dopaminergic neuron cells was conducted in both the ventral tegmental area (VTA) and the substantia nigra par compacta (SNpc). The bar graph depicting the PD group demonstrated a significant difference in the number of TH‐positive neurons, with a count of 303.40 ± 40.12, significantly lower compared to the NC group at 451.40 ± 9.432 and the HP group at 444.60 ± 28.26 (*p = .015, *p = .022, respectively), as indicated in the sample size of *n* = 5 mice per group.

The immunoreactivity of TH in the striatum of the nigrostriatal pathway was observed (Figure 4a). The results of this study demonstrate a significant reduction in the optical density of TH immunoreactivity in the striatum of the nigrostriatal pathway within the PD group compared to the NC and HP groups, as illustrated in Figure 4d (PD group 0.11 ± 0.019, NC 0.37 ± 0.029, HP 0.35 ± 0.029, ***p < .001, respectively). Furthermore, the optical density of the LP group (0.12 ± 0.018) and FP group (0.14 ± 0.028) was also significantly lower than that of the NC group (***p < .001, **p < .01, respectively). In contrast, the optical density of the MP group (0.21 ± 0.05) exhibited only a slight difference from the NC group (*p = .044). The sample size for the NC group was 3, while the sample sizes for the PD, LP, MP, HP, and FP groups were 5 each.

#### 3.7.2 Enteric nervous system in the colon expresses α‐synuclein

In this investigation, the expression of alpha‐synuclein was predominantly observed within the inner circular and outer longitudinal smooth muscle layers of the colon. Notably, it was also evident in the myenteric plexus, situated amidst the smooth muscle layers. The identification of α‐synuclein expression was detected through brown deposits, visualized using 3,3’‐diaminobenzidine (DAB) chromogen, and counterstained with hematoxylin (blue), in Figure 4b. Quantification of α‐synuclein expression in colon tissues was accomplished using the H‐score, determined through the Qupath software, as illustrated in Figure 4e. The bar graph elucidates a significant increase in α‐synuclein H‐score within the PD group (179.1 ± 22.06) compared to the NC group (12.58 ± 2.876) (**p < .01). Furthermore, a notable difference was observed between the NC and LP groups (151.3 ± 29.23) (*p = .039).

### 3.8 Western blot analysis of brain and colon tissue in a PD mouse model

#### 3.8.1 Total TH protein expression in the nigrostriatal tissue of mouse brains

**FIGURE 5:**
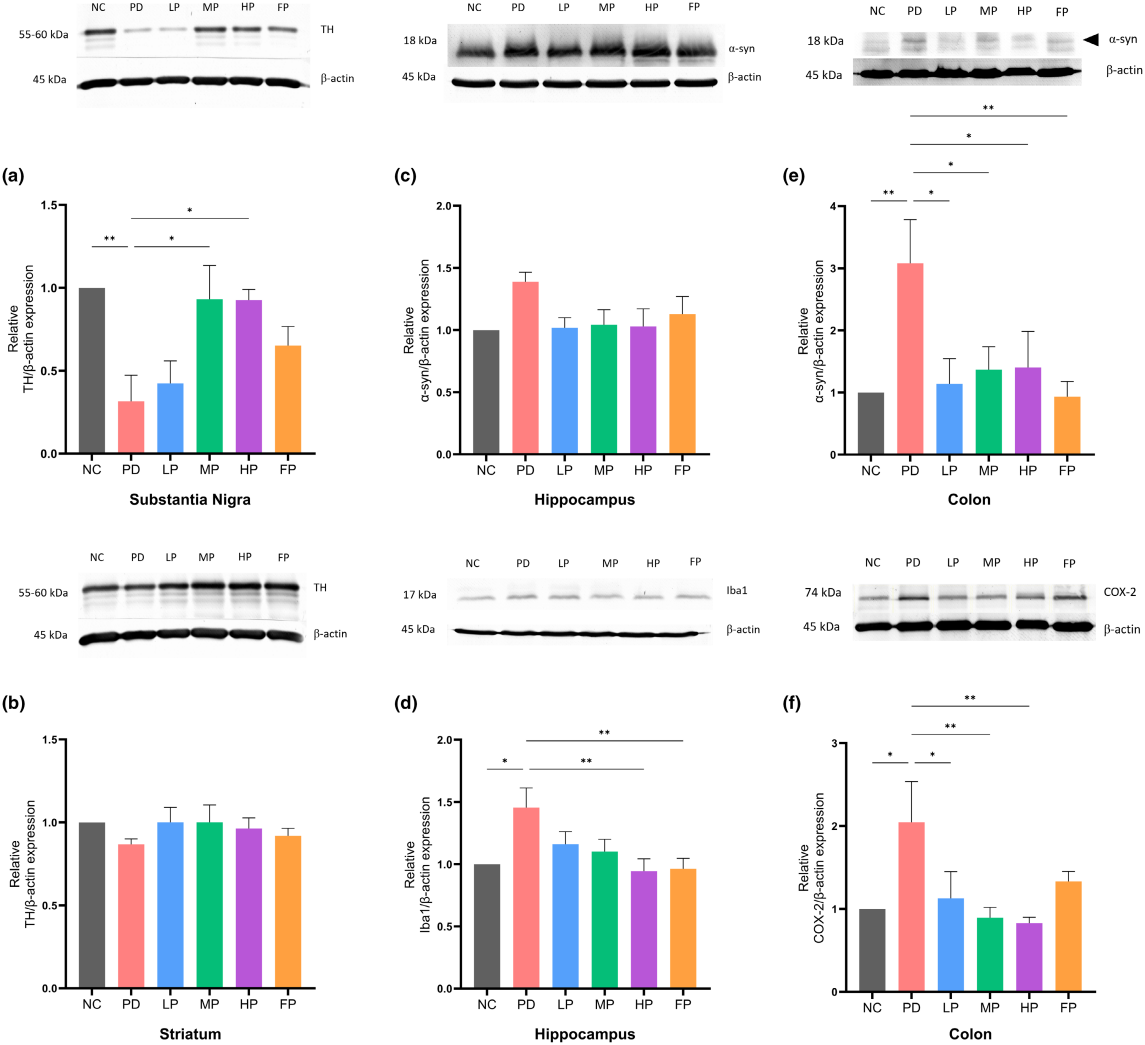
Western blot analysis and relative expression levels of tyrosine hydroxylase (TH) in substantia nigra (a) and striatum (b) homogenates; relative expression levels of α‐synuclein (α‐syn) and ionized calcium‐binding adaptor molecule 1 (Iba1) in hippocampus homogenates (c and d); and relative expression levels of α‐syn and COX‐2 in mouse colon homogenates (e and f). The data were analyzed by one‐way ANOVA followed by the Dunnett test and are expressed as mean ± SEM (*n* = 4–7 for substantia nigra and striatum homogenates and *n* = 5–6 for hippocampus and colon homogenates). Asterisks indicate significant differences (*p < .05, **p < .01, ***p < .001).

Figure 5 represents the protein bands derived from the Western blot analysis, accompanied by the corresponding bar graph delineating the levels of tyrosine hydroxylase (TH) expression in the substantia nigra (SN) and striatum (ST) brain tissues; discernible trends emerge. Specifically, within the substantia nigra brain tissue area (Figure 5a), the PD group exhibited a significant reduction in the relative expression of TH/β‐actin in comparison to the NC group (**p = .004), PD vs. MP (*p = .012), and PD vs. HP (*p = .011).

However, no statistically significant difference in TH expression levels was observed in the striatum region (Figure 5b). Nonetheless, within this context, the PD group does exhibit marginally lower TH levels relative to all other groups in this region.

#### 3.8.2 Protein expression levels of α‐synuclein and Iba1 in the hippocampus

As illustrated in Figure 5c, although there was no significant difference observed in the α‐synuclein expression levels in this region, it was noted that the PD group exhibited the highest expression level of α‐synuclein compared to the other groups.

Furthermore, an assessment of Iba1 expression levels in the hippocampus region (Figure 5d) implicated it in immune response and inflammation. The findings revealed an increased upregulation of Iba1 expression in the PD group as compared to the NC, HP, and FP groups (*p = .013, **p = .007, **p = .006, respectively).

#### 3.8.3 Protein expression levels of α‐synuclein and COX‐2 in the colon

Figure 5e shows that α‐synuclein expression exhibited a statistically significant increase in the PD group when compared to all other groups, namely the NC, LP, MP, HP, and FP groups (**p = .007, *p = .027, *p = .041, *p = .046, **p = .008, respectively).

Furthermore, the enzyme COX‐2 (Figure 5f), induced during inflammatory responses, demonstrated a significant increase in the PD group compared to all other groups, including the NC, LP, MP, and HP groups (**p = .012, *p = .037, *p = .005, *p = .004, respectively).

### 3.9 Gut microbiome analysis

#### 3.9.1 Alpha diversity analysis of the bacterial community

**FIGURE 6:**
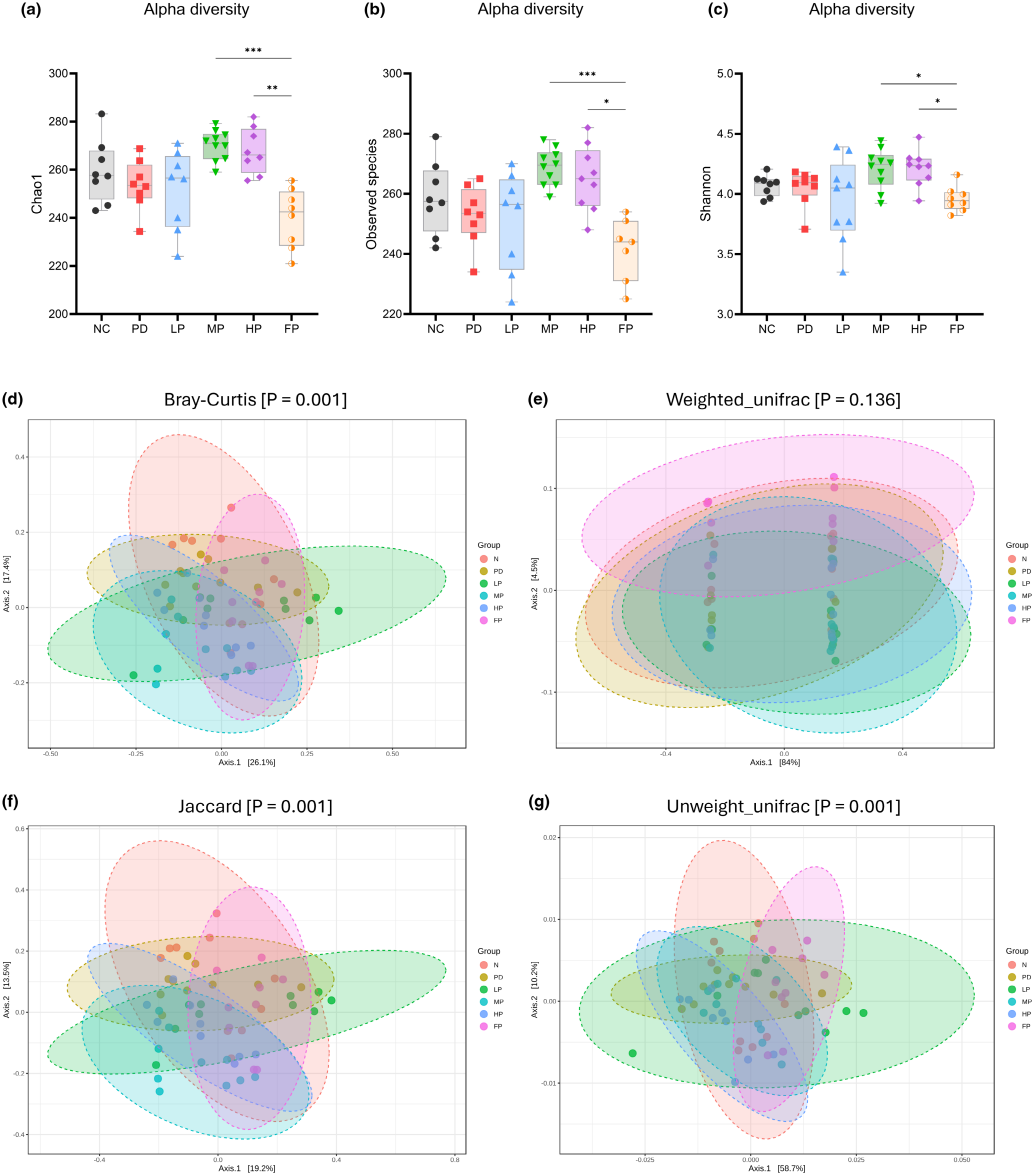
Box and whisker plots indicating Chao1 (a), observed (b), and Shannon (c) alpha diversity indices, as well as PCoA plots for Bray‐Curtis (d), weighted unifrac (e), Jaccard (f), and unweighted unifrac (g) beta diversity metrics. Kruskal–Wallis was performed for alpha diversity analysis (*n* = 8–10 per group), and PERMANOVA with 999 permutations was used for beta diversity analysis (*n* = 9–10 per group). Asterisks indicate significant differences (*p < .05, **p < .01, ***p < .001).

The study results revealed that the MP and HP groups exhibited the highest values for Chao1, observed species, and Shannon indexes, denoting increased fecal bacterial species richness and diversity within the perilla seed oil diet treatment groups (Figure 6a–c). The observed species, Chao1, and Shannon diversity indexes pertaining to the caecum in the MP and HP groups demonstrated a statistically significant increase compared with those in the FP group (*p < .05, **p < .01, ***p < .001). Conversely, no significant differences between the richness of caecum bacteria content in PD and other groups were observed.

#### 3.9.2 Beta diversity analysis between different environmental communities

The investigation into the structural composition of bacterial communities among distinct sample groups involved the utilization of beta diversity indices, namely Bray‐Curtis, Weighted unifrac, Jaccard, and Unweighted unifrac (Figure 6d–g). The statistical analysis revealed that only the Bray‐Curtis, Jaccard and Unweighted_unifrac indexes exhibited a significant differentiation in microbial composition among the six distinct groups of cecal contents sampled from mice (*p* = .001).

#### 3.9.3 Analysis of bacterial taxonomic composition

**FIGURE 7:**
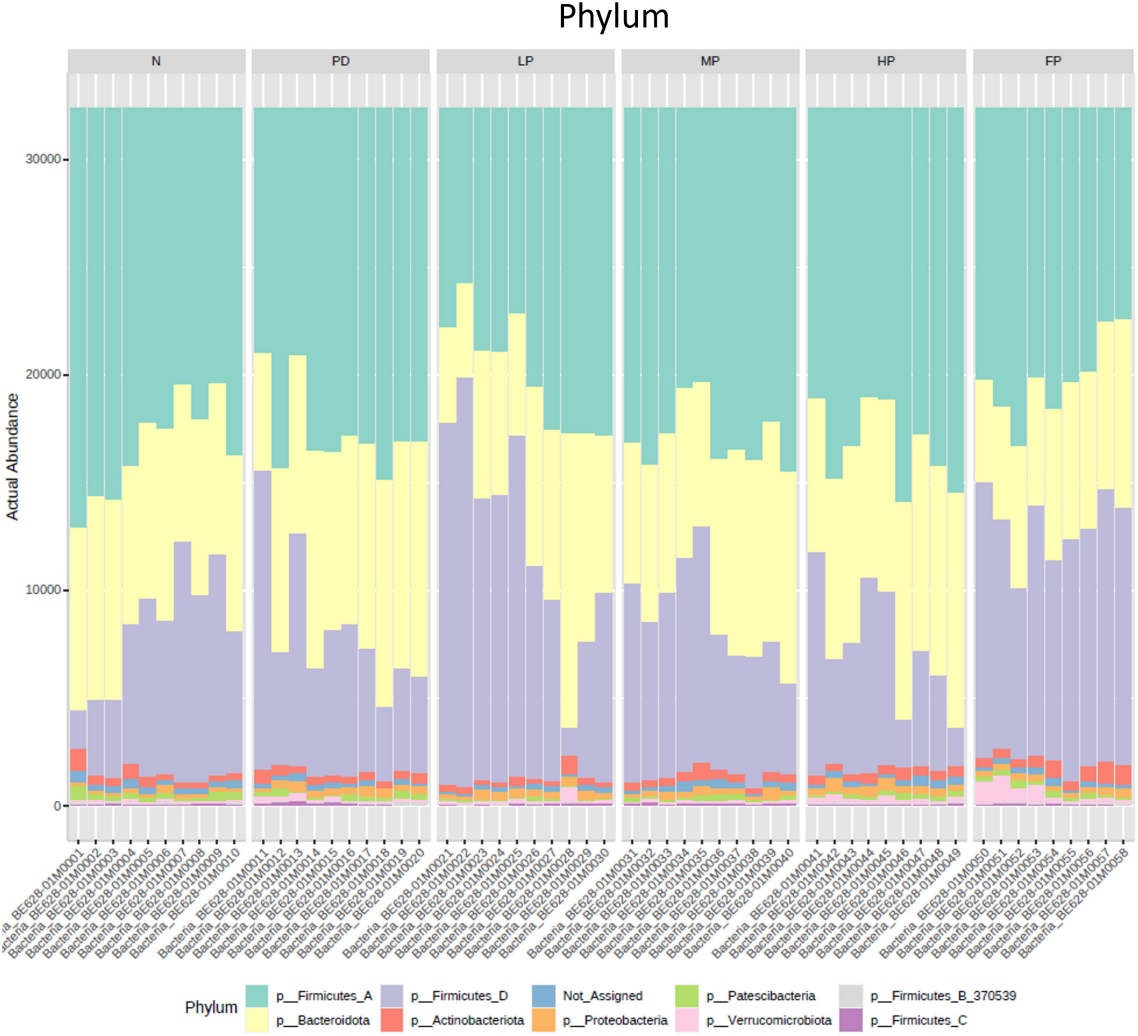
Analysis of bacterial composition in the caecum contents of mice. Taxonomy community analysis at the phyla level.

A total of 10 bacterial phyla were identified based on the actual abundance of the gut microbiota, as depicted in the histogram (Figure 7). The prevalent phyla in terms of abundance across all samples included *Firmicutes*, *Bacteroidota*, *Actinobacteriota*, *Proteobacteria, Patescibacteria*, and *Verrucomicrobiota*, respectively.

At the family level, the composition of gut microbiota exhibited consistent patterns across all samples. The majority of actual abundances at the family level were observed to be *Lachnospiraceae*, *Muribaculaceae*, *Lactobacillaceae*, *Bacteroidaceae*, *Erysipelotrichaceae*, and *Oscillospiraceae_88309*, respectively (data not shown).

An investigation into alterations in gut microbiota abundance at the genus level was conducted, considering the impact of both rotenone‐induced Parkinson's disease and dietary interventions employing middle doses of perilla oil (MP) or fish oil (FP). The dominant actual abundances at the genus level were identified as *CAG_95*, *Ligilactobacillus*, *UBA7173*, *Dubosiella*, *Kineothrix*, *Alloprevotella*, and *Lactobacillus*, respectively (data not shown).

#### 3.9.4 Impact of perilla seed oil and fish oil diets on the composition of mouse intestinal microbiota

**FIGURE 8:**
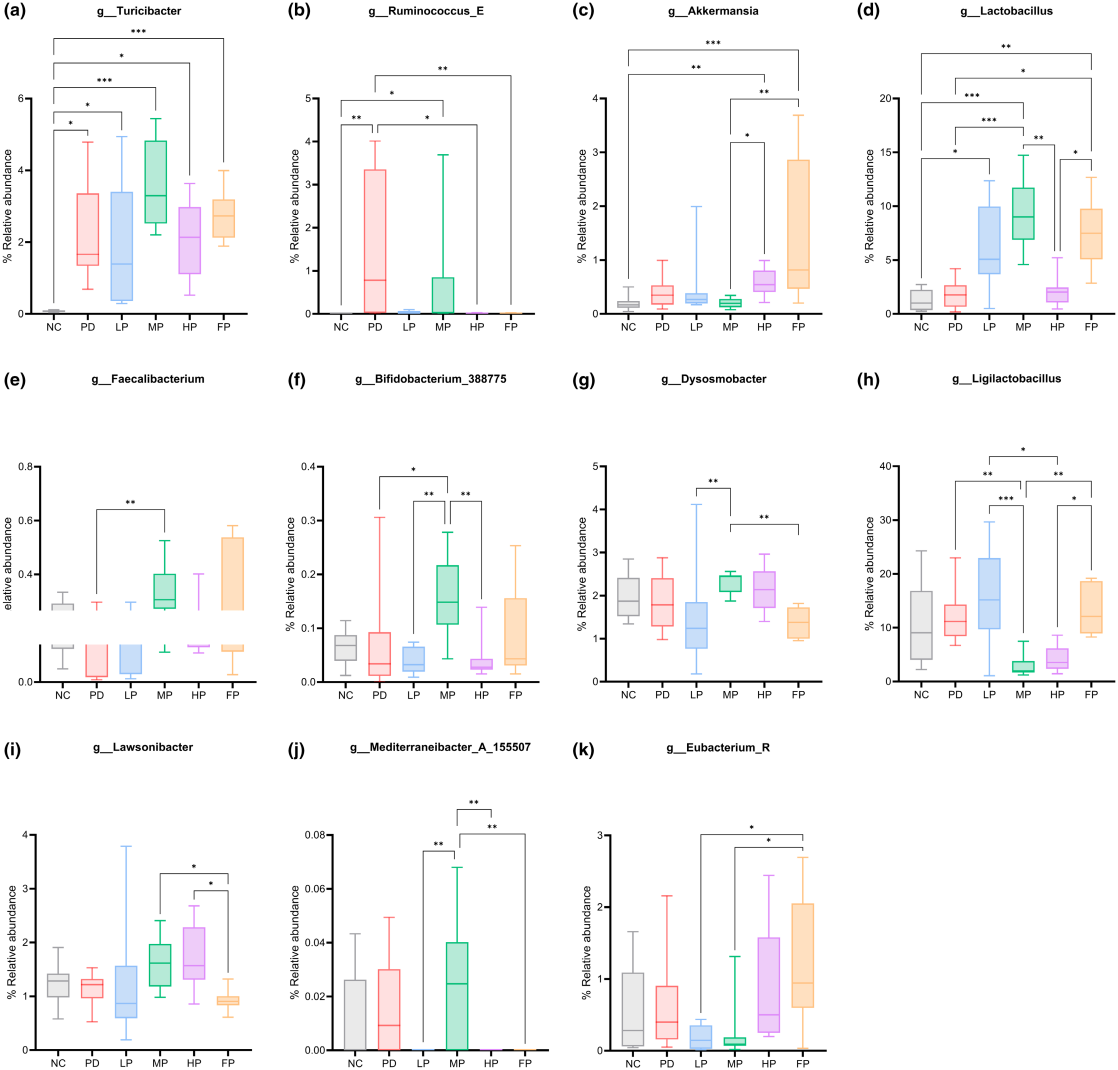
The relative abundance of *Turicibacter* (a), *Ruminococcus_E* (b), *Akkermansia* (c), *Lactobacillus* (d), *Faecalibacterium* (e), *Bifidobacterium_388775* (f), *Dysosmobacter* (g), *Ligilactobacillus* (h), *Lawsonibacter* (i), *Mediterraneibacter_A_155507* (j), and *Eubacterium_R* (k) in the cecum, affected by dietary treatment in a rotenone‐induced Parkinson's disease model in mice. Each group consisted of 9–10 mice. Data were analyzed using the Kruskal–Wallis test, followed by Dunn's pairwise multiple comparisons. The results are presented as boxes and whiskers, median, and IQR, with asterisks indicating significant differences (*p < .05, **p < .01, ***p < .001).

At the genus level, among the 46 identified genera, 11 exhibited significant alterations in abundance attributable to the influence of dietary oil treatments in the context of rotenone‐induced Parkinson's disease. These 11 genera exerted discernible effects on the intestinal microbiota community across various mouse groups, as illustrated in Figure 8a–k. In Figure 8a, the *Turicibacter* genus, classified under the *Turicibacteraceae* family within the *Firmicutes* phylum, exhibited a significant increase in abundance across all rotenone‐induced groups, excluding the NC (non‐induced) group. The statistical comparisons are as follows: NC vs. PD (*p = .013), NC vs. LP (*p = .047), NC vs. MP (***p < .001), NC vs. HP (*p = .023), and NC vs. FP (***p < .001). Similarly, in Figure 8b, a comparison with the PD group reveals a substantial increase in the abundance of *Ruminococcus_E* within the *Firmicutes_A* phylum, *Ruminococcaceae* family. The statistical comparisons are as follows: NC vs. PD (*p = .003), NC vs. MP (*p = .021). These figures elucidate the association with rotenone‐induced Parkinson's disease.

In Figure 8c–k, the microbial composition analysis revealed that the abundance of *Akkermansia*, *Lactobacillus*, *Faecallibacterium, Bifidobacterium*, *Dysosmobacter, Ligilactobacillus*, *Lawsonibacter, Mediterraneibacter_A_155507*, and *Eubacterium_R* was notably elevated in mice subjected to perilla oil treatment, particularly in the MP group, as well as the HP, LP, and FP groups. Specifically, the relative abundance of the *Akkermansia* genus within the *Verrucomicrobiota* phylum, *Akkermansiaceae* family, was significantly reversed by a fish oil diet in the FP group, as evidenced by the marked difference between the NC and FP groups (***p < .001). Remarkably, the middle dose of the perilla seed oil (MP) diet exhibited a significant augmenting effect on the abundance of the *Lactobacillus* genus within the *Firmicutes* phylum, *Lactobacillaceae* family. Statistical comparisons indicated substantial differences between NC vs. MP (***p < .001), PD vs. MP (***p < .001), NC vs. FP (***p < .001), and PD vs. FP (*p = .016).

## 4 DISCUSSION

Our study confirms previous findings that perilla seed oil contains a high percentage (51% to 64%) of omega‐3 polyunsaturated fatty acid α‐linolenic acid (ALA) (Ding et al., 2012; Gwari et al., 2014; Peiretti, 2011; Shin & Kim, 1994). We determined the total tocopherol content and phenolic and flavonoid compounds and estimated the in vitro antioxidant capacity using ORAC and FRAP assays. Our results align with prior research, showing that crude oil has the highest antioxidant capacity, which remains consistent throughout the oil extraction process (Pan et al., 2020).

### 4.1 Effect of edible oil diets on body weight and food intake in a Parkinson's disease mouse model

The high‐dose perilla seed oil diet (20% perilla oil) administered to the HP group in our Parkinson's disease mouse model resulted in reduced food intake, likely due to satiety. Conversely, the FP group (10% fish oil diet) showed lower body weight but higher food intake, indicating the fish oil diet's impact on appetite. Fish oil is associated with metabolic benefits such as improved glucose intolerance, reduced food intake, mitigation of anxiety and depression‐related behaviors, and anti‐inflammatory responses (Demers et al., 2020; Du et al., 2015).

### 4.2 Effect of dietary oils on non‐motor symptoms and gastrointestinal dysfunction in the rotenone‐induced Parkinson's disease mouse model

Braak's proposed staging of Parkinson's disease (PD), which suggests the presence of non‐motor symptoms such as constipation, olfactory impairment, depression, anxiety, and sleep disturbance, is present (Rietdijk et al., 2017). Gastrointestinal dysfunction, a prevalent non‐motor symptom in Parkinson's disease (PD), is evident in our rotenone‐induced PD mouse model with diminished colon length (Chu et al., 2023; Zhao et al., 2021). This is likely due to disrupted neural pathways affecting gut function. Interestingly, perilla seed oil supplementation increased colon length in mice on a high‐fat diet (Thomas et al., 2020a). We observed similar increases in colon length in groups receiving middle‐ (MP) and high‐dose (HP) perilla seed oil, suggesting its potential therapeutic efficacy on the colon.

### 4.3 Impact of oils on neurobehavioral motor function and anxiety‐like symptoms in a rotenone‐induced Parkinson's disease mouse model

Our study initiates the exploration of perilla seed oil's impact on motor impairment in rotenone‐induced Parkinson's disease. Previous studies have reported the potential of perilla seed oil, rich in omega‐3 fatty acids, to enhance cognitive function (Kaewwongse et al., 2019).

The present study used the pole test, a common assessment for “bradykinesia” in Parkinson's disease (PD), to evaluate motor impairments. Consistent with previous studies (Chu et al., 2023), rotenone‐induced motor impairment was observed in the PD group. However, a diet with perilla seed oil significantly reversed this impairment, particularly in the MP and HP groups.

The cylinder test in our study revealed that rotenone‐induced Parkinson's disease (PD) mice exhibited fewer rearing behaviors, indicating motor deficits (Miyazaki et al., 2020). However, high‐dose perilla (HP) and fish oil (FP) groups exhibited similar rear‐up behavior to the control (NC) group.

In the open‐field test, the PD group showed more freezing behavior and less locomotion, indicating anxiety‐like behavior and impaired locomotor function. Conversely, the LP, MP, and HP groups, which received high‐dose perilla seed oil treatment, showed reduced freezing and more locomotion. The PD group also made fewer entries into the central area, a behavior linked to anxiety in Parkinson's disease due to its impact on the brain's dopamine system (Seibenhener & Wooten, 2015; Sestakova et al., 2013).

Our study found that the PD group showed reduced rearing behaviors and increased grooming behaviors, consistent with anxiety‐like behavior and stress (Seibenhener & Wooten, 2015). This contrasts with other models of Parkinson's and Alzheimer's diseases, which show decreased grooming behavior. The increased grooming in the PD group, associated with the impairment of the nigrostriatal dopaminergic system and dopamine depletion, suggests a distinct manifestation of anxiety‐related behavior in Parkinson's disease (Kalueff et al., 2016; Sharghi et al., 2023).

### 4.4 Therapeutic potential of perilla seed oil: Reversing protein expression alterations and modulating the gut–brain axis in a Parkinson's disease model

Rotenone injection in the PD group resulted in reduced tyrosine hydroxylase (TH‐positive cells) in the substantia nigra (SN) and striatum (ST) of mice, indicative of dopaminergic neuron degeneration, as indicated by immunohistochemistry results. This is consistent with a previous study that employed the rotenone model to induce PD through intrastriatal and oral administration, alongside dietary interventions incorporating fish oil rich in EPA and DHA PUFA co‐supplements with uridine (Perez‐Pardo et al., 2018), prebiotic fiber (Perez‐Pardo, De Jong et al., 2017), or vitamin E (Taghizadeh et al., 2017). However, treatment with high‐dose perilla seed oil (HP) reversed this loss, suggesting its potential as a neuroprotective property.

Our study assessed tyrosine hydroxylase (TH) protein expression in dopamine‐containing cells within the nigrostriatal pathway using Western blot analysis. We observed a significant reduction in TH protein expression in the PD group, consistent with previous studies (Jia Liu et al., 2018). However, treatment with middle‐ (MP) and high‐dose (HP) perilla seed oil increased TH protein expression. This may be linked to the conversion of alpha‐linolenic acid (ALA), an essential omega‐3 fatty acid in perilla seed oil, to docosahexaenoic acid (DHA), known for its neuroprotective properties (Avallone et al., 2019; Chappus‐McCendie et al., 2019; de Bus et al., 2019).

Previous studies indicate that a diet rich in PUFAs, including EPA, DHA, and ALA, can potentially alleviate Parkinson's and Alzheimer's diseases (Avallone et al., 2019; Canhada et al., 2018). Our study found that perilla seed oil, rich in ALA, may enhance DHA synthesis, leading to increased TH protein expression and the potential preservation of dopaminergic neurons, thereby potentially alleviating motor impairments in Parkinson's disease.

This study investigated the gut–brain axis and its association with α‐synuclein (α‐syn) presence in the gut and brain, contributing to Parkinson's disease progression. We identified α‐synuclein in the myenteric plexus of rotenone‐induced Parkinson's disease mice, underscoring the importance of this gut–brain communication pathway (Carabotti et al., 2015). In line with previous studies (Perez‐Pardo et al., 2018), our research found that perilla seed oil treatment at high and middle doses reduced α‐synuclein accumulation in the colon myenteric plexus in rotenone‐induced Parkinson's disease. This suggests that perilla seed oil may protect against α‐synuclein aggregation in the gut, potentially slowing the spread of pathology to the brain (Chao et al., 2020; Klann et al., 2022).

In our study, we examined protein expressions in the hippocampus, specifically alpha‐synuclein and microglia activation (Iba1). The Western blot results showed elevated alpha‐synuclein and Iba1 expressions in Parkinson's disease (PD) mice, suggesting disease pathology and neuroinflammation. However, treatment with high dose perilla seed oil and fish oil attenuated both alpha‐synuclein and microglia activation.

Alpha‐synuclein, a key component of Lewy bodies in Parkinson's disease (PD), accumulates in specific brain regions like the hippocampus (Villar‐Conde et al., 2021). Studies suggest that α‐synuclein pathology propagates from the intestinal colon to higher‐order brain regions via the enteric nervous system (Rietdijk et al., 2017). The hippocampus, characterized by complex synaptic connections, plays a crucial role in cognitive functions such as learning, emotions, and memory (Villar‐Conde et al., 2021; Wheeler et al., 2015).

Pathological α‐synuclein in the hippocampus is linked to cognitive impairment and dementia in diseases like Lewy body dementia and Parkinson's disease (Guo et al., 2022). Pathological α‐synuclein accumulates in excitatory presynaptic terminals (Nwabufo & Aigbogun, 2022), contributing to neuronal death. This, along with microglial activation and inflammatory processes induced by synucleinopathies, triggers inflammatory cascades not only in the hippocampus but also in dopaminergic neurons in the substantia nigra. This protein accumulates in excitatory presynaptic terminals, contributing to neuronal death and triggering inflammatory cascades in the hippocampus and dopaminergic neurons in the substantia nigra (Booms & Coetzee, 2021; Cardinale et al., 2021; Guo et al., 2022). Animal models show that α‐synuclein spread is associated with Parkinson's disease progression and motor dysfunction. Perilla seed oil and fish oil may reduce α‐synuclein overexpression and mitigate microglial inflammation, offering neuroprotective effects.

Braak's hypothesis posits that an unknown pathogen, potentially ingested, could induce α‐synuclein aggregation in enteric nerves, leading to PD. Braak's hypothesis suggests that an unknown pathogen, potentially a bacterium or virus, ingested through the gastrointestinal tract may be implicated in sporadic PD (Braak et al., 2003; Jan et al., 2021).

Increased cox‐2 expression in the colon, a response to inflammatory stimuli, may contribute to local inflammation and PD progression. Omega‐3 fatty acids, beneficial for gastrointestinal health, may mitigate this inflammation (Kangwan, Pintha et al., 2021). Omega‐3 fatty acids, found in perilla oil and fish oil, have anti‐inflammatory properties. They can modulate the production of inflammatory mediators and promote the generation of anti‐inflammatory molecules. Omega‐3 polyunsaturated fatty acids (PUFAs) exhibit anti‐inflammatory properties by competing with omega‐6 PUFAs, reducing the production of pro‐inflammatory eicosanoids. Eicosapentaenoic acid (EPA), an omega‐3 PUFA, can generate alternative eicosanoids that exhibit reduced pro‐inflammatory activity. Omega‐3 PUFAs also facilitate the resolution of inflammation by serving as precursors for “specialized pro‐resolving mediators” (SPMs). These SPMs, including resolvins, protectins, and maresins, contribute to the resolution of inflammation and the restoration of body homeostasis (Barnig et al., 2019; Simonetto et al., 2019).

Omega‐3 PUFA, like NSAIDs, competitively inhibit COX‐2 enzyme activity, leading to increased production of anti‐inflammatory eicosanoids, particularly protectins and resolvins (Lee et al., 2009; Norling & Perretti, 2013). Thus, incorporating omega‐3 PUFA into the diet can have prolonged anti‐inflammatory effects, offering a potential mild anti‐inflammatory intervention. In contrast, NSAIDs act more immediately by directly inhibiting enzymatic activity (Ye & Ghosh, 2018).

### 4.5 Gut microbiota composition related to Parkinson's disease

Research suggests that diet significantly influences the gut microbiota, which is linked to the development of Parkinson's disease (PD) through the gut–brain axis (Jan et al., 2021; Perez‐Pardo, Kliest et al., 2017).

The *Turicibacter* genus has been positively correlated with PD, with higher abundance associated with clinical features of PD, showing correlations with disease severity, medication, and non‐motor symptoms (Jin et al., 2019; Salim et al., 2022). PD patients have been found to have reduced levels of short‐chain fatty acids (SCFA)‐producing bacteria and altered gut microbiota composition (Nair et al., 2018). Although the *Akkermansia* genus is known for SCFA production, other studies have reported an increased abundance of *Akkermansia* in PD patients' feces (Lorente‐Picón & Laguna, 2021), associated with a proinflammatory condition leading to heightened gut permeability in PD. (Nie et al., 2022; Parada Venegas et al., 2019). Additionally, decreased *Prevotella* and *Ruminococcus*, along with increased *Akkermansia*, have been observed in PD patients (Bullich et al., 2019). Studies align with our findings, showing a rise in *Akkermansia* and a decrease in *Prevotella* and *Ruminococcus* in the FP group. Interestingly, *Akkermansia muciniphila*, known for stimulating mucus synthesis and promoting mucosal layer adhesion, was implicated. However, there is an imbalanced increase in *Akkermansia muciniphila*, *Prevotella*, and *Ruminococcus*, all mucin‐degrading bacteria. Its presence predicts increased intestinal permeability and a leaky gut (Martín et al., 2023), which may be linked to neurological disorders, including PD (Bullich et al., 2019; Fang et al., 2021; Glover et al., 2022). Meta‐analyses report increased *Akkermansia* and reduced *Roseburia* and *Faecalibacterium* in PD patients, recognized as SCFA producers (Lorente‐Picón & Laguna, 2021). Reduced SCFAs may be associated with neuroinflammation in PD (Boktor et al., 2022; Lorente‐Picón & Laguna, 2021; Nishiwaki, Ito, et al., 2022). In this study, the PD mice group displayed reduced *Roseburia* and *Faecalibacterium*, but *Faecalibacterium* increased in the MP and FP groups.

Research has shown a significant relationship between the *Ruminococcus* and *Akkermansia* genera, with an increased abundance of *Ruminococcus torques* and *Collinsella* observed in patients with dementia with Lewy bodies (DLB), indicating the onset of Parkinson's disease (PD) (Nishiwaki, Ueyama, et al., 2022). *Ruminococcus*, a genus of the *Firmicutes* phylum, is known for its production of short‐chain fatty acids (SCFAs) with neuroprotective effects on dopaminergic neurons. However, its role in mucin degradation can lead to inflammation in the colon (Hamamah et al., 2022). This study found a higher abundance of *Ruminococcus* and *Collinsella* in the PD mouse group, while *Ruminococcus* was nearly absent in the normal control (NC) and FP groups. A recent study assessed the effects of *Ruminococcus* on dopaminergic neurons in mice induced with MPTP neurotoxicity, which disrupts gut microbiota balance and leads to an increased concentration of *Ruminococcus* and degeneration of tyrosine hydroxylase‐positive cells in the nigrostriatal pathway. Consumption of Korean red ginseng by MPTP‐treated mice in a PD model reduced the degeneration of dopaminergic neurons by decreasing the abundance of *Ruminococcus*, leading to increased tyrosine hydroxylase activity and elevated dopamine levels (Jeon et al., 2021). Our results align with previous findings, which suggested that the abundance of *Turicibacter*, *Ruminococcus*, *Akkermansia*, and *Faecalibacterium* is associated with rotenone‐induced Parkinson's disease.

*Lactobacillus* and *Bifidobacteria* (Chu et al., 2023; Kong et al., 2022; Tian et al., 2020), commonly used probiotics, have been shown to alleviate gastrointestinal dysfunction in Parkinson's disease (PD) patients and animal models (Zhu et al., 2022). Specifically, probiotic supplementation, particularly with *Lactobacillus*, has been posited as a psychobiotic intervention in the context of neurodegenerative diseases (Cheng et al., 2019). It reduces α‐synuclein aggregation, improves oxidative damage resistance, suppresses neuroinflammation and immune cell activity, modulates glial cell hyperactivation, promotes intestinal motility, relieves constipation, and ameliorates motor deficits and neurotoxicity (Chen et al., 2022; Liu et al., 2016; Ma et al., 2023; Zhao et al., 2021). A decline in neurotransmitter‐producing bacteria, including *Bacillus* spp., *Lactobacillus* spp., and *Streptococcus* spp., has been observed in PD patients, suggesting a link between gut microbial composition, neurotransmitter production, and PD pathophysiology (Jing Liu et al., 2020). This modulation of neurotransmitter levels by gut bacteria could provide a new approach for managing non‐motor PD symptoms (Chen et al., 2021). Microbial treatments, particularly with *Lactobacillus* spp. and *Bifidobacterium* spp., have shown promise in treating PD symptoms, improving gut motility, alleviating constipation, abdominal pain, and bloating, and enhancing stool consistency (Gazerani, 2019; Tan et al., 2021).

This study found a notable presence of *Lactobacillus* in the MP, FP, and LP groups, with a depletion in the rotenone‐induced PD mouse model.

Significant decreases in butyrate‐producing microorganisms, particularly *Lawsonibacter* (Sakamoto et al., 2018), were observed, except in the HP and MP groups. Stroke patients showed altered microbiome diversity and composition, with a depletion of beneficial gut bacteria, *Lawsonibacter* and *Kineothrix* (Hammond et al., 2022). Similarly, a decrease in *Lawsonibacter* and *Kineothrix* was found in a 5‐month‐old PAP transgenic mouse Alzheimer's disease (AD) model (Li et al., 2020). This study revealed an increase in *Kineothrix* in the NC group, suggesting that the reduction in butyrate‐producing bacteria, including *Lawsonibacter* and *Kineothrix*, may be associated with AD (Haas, 2017; Sakamoto et al., 2018).

### 4.6 Potential mechanism of omega‐3 fatty acids in the gut microbiota

Beneficial bacteria such as *Akkermansia*, *Lactobacillus*, and *Bifidobacterium* are known for their ability to produce short‐chain fatty acids (SCFAs) (Nogal et al., 2021). Moreover, *Akkermansia*, in particular, has been highlighted for its role in obesity modulation through probiotic supplementation, improving inflammation, preventing metabolic disorders, and enhancing insulin sensitivity and glucose homeostasis (Abuqwider et al., 2021; Costantini et al., 2017). Mice subjected to fecal transplantation of gut microbes associated with a fish oil‐rich diet showed an increased abundance of *Akkermansia muciniphila*, known for mitigating weight gain and improving glucose metabolism. Compared to mice receiving gut microbiota transplantation from mice fed with lard fat, the fish oil group showed improved inflammation biomarkers and reduced weight gain (Caesar et al., 2015). This study aligns with these findings, showing a significant increase in *Akkermansia* in the FP group and maintenance of weight gain over a 6‐week period, suggesting potential metabolic benefits of including fish oil in the diet.

The newly discovered bacterium, *Dysosmobacter welbionis J115*, known for its butyrate production, was isolated from the human gut, including those with metabolic syndrome (Le Roy et al., 2022). Notably, this correlation was most pronounced in the high‐abundance *Dysosmobacter* populations within the MP and HP groups. Given these findings, *Dysosmobacter welbionis J115* emerges as a promising candidate for probiotic intervention targeted at weight maintenance.

Omega‐3‐rich diets are linked to an increased presence of SCFA‐producing bacteria, specifically *Lactobacillus* and *Bifidobacterium*, in the gut (Nogal et al., 2021). This study suggests that variations in bacterial abundance may be tied to the different amounts and types of edible oil treatments given to each group. Notably, the MP group had the highest abundance of *Lactobacillus* and *Bifidobacterium*. Previous research showed that perilla seed oil (PSO) treatment alleviated gut dysbiosis caused by a high‐fat diet (HFD) in rats with obesity and insulin resistance, reducing intestinal and systemic inflammation and metabolic disturbances (Kangwan, Pratchayasakul et al., 2021). Similarly, long‐term consumption of a high‐fat diet led to obesity and colon inflammation in C57BL/6J mice, but supplementation with perilla seed oil increased the abundance of *Bifidobacteria* (Thomas et al., 2020b).

Omega‐3 PUFA supplementation enhances the abundance of *Bifidobacterium*, impacting omega‐3 PUFA metabolism and absorption and promoting short‐chain fatty acids (SCFAs) that benefit gut health. ALA‐enriched diets also influence the gut microbiota, with *Bifidobacterium* strains metabolizing ALA (Fu et al., 2021).

Specific *Bifidobacterium* strains can convert free linolenic acid and linoleic acid into conjugated linolenic acid (CLNA) and conjugated linoleic acid (CLA) isomers through a biohydrogenation process in the gut (Gorissen et al., 2010). These conjugated fatty acids have shown beneficial effects, including body weight regulation, anticarcinogenic activity, lipid metabolism modulation, and antioxidant and anti‐inflammatory activities (Basak & Duttaroy, 2020; Carina Paola Van et al., 2012).

*Ligilactobacillus*, a new name‐based *Lactobacillus*, shows potential as an alternative probiotic for oral administration, alongside *Lactobacillus* and *Bifidobacterium* strains. It can mitigate intestinal pathogen invasion, prevent chronic inflammation, modulate cytokine levels, restore intestinal barrier integrity, and enhance gut microbiota homeostasis (Quilodrán‐Vega et al., 2020; Yao et al., 2021). This study found a significant increase in *Ligilactobacillus* in the LP and FP groups.

*Eubacterium*, a butyrate‐producing bacterium, increased significantly after a 2‐week omega‐3‐rich diet (Noriega et al., 2016; Wellington et al., 2022). Replacing soybean oil, palm oil, and beef tallow with fish oil in the diet of tiger puffer fish also increased *Eubacterium* abundance (Kong et al., 2023). The FP group in this study showed a similar increase in *Eubacterium*. This suggests that omega‐3 PUFA may increase specific butyrate‐producing bacteria, benefiting intestinal health, serving as an energy source for the colonic mucosa, and regulating gene expression, inflammation, differentiation, and apoptosis in host cells (Louis & Flint, 2009).

This study found that *Mediterraneibacter*, a member of the *Lachnospiraceae* family within the *Firmicutes* phylum (Wongkuna et al., 2021), was most abundant in the MP group, followed by the PD and NC groups, and was absent in the LP, HP, and FP groups. Previous research showed that *Mediterraneibacter*, found in probiotic‐fermented milk with passion fruit pulp and either palm or buriti pulp, has a beneficial association with human intestinal health. It produces short‐chain fatty acids (SCFAs) such as lactic acid, formic acid, and acetic acid from carbohydrate metabolism and uses these organic acids to produce butyrate. The increased abundance of *Mediterraneibacter*, along with its SCFA production, is considered a health‐promoting biomarker (Borgonovi et al., 2022).

## 5 CONCLUSION

Perilla seed oil offers neuroprotective benefits through various mechanisms. It is enriched with bioactive compounds such as omega‐3 polyunsaturated fatty acids, particularly alpha‐linolenic acid (ALA), and has inherent antioxidant properties. High doses (HP) of perilla seed oil demonstrate efficacy in alleviating both motor and non‐motor symptoms of Parkinson's disease by reducing dopaminergic neuronal loss, upregulating tyrosine hydroxylase (TH), suppressing α‐synuclein and inflammatory responses, and modulating the gut–brain axis. Perilla seed oil also enhances gut microbiota diversity, with the middle dose (MP) showing the most efficacy. These findings suggest that consuming ALA, an omega‐3 fatty acid from perilla oil, may positively influence gut microbiome composition in individuals with Parkinson's disease. Further research is imperative to elucidate precise therapeutic mechanisms and ascertain the optimal dosage required to maximize these benefits.

## AUTHOR CONTRIBUTIONS

**Peerapa Techaniyom:** Conceptualization (lead); data curation (lead); formal analysis (lead); funding acquisition (lead); investigation (lead); methodology (lead); project administration (lead); software (equal); validation (equal); visualization (lead); writing – original draft (lead); writing – review and editing (equal). **Chawin Korsirikoon:** Data curation (equal); investigation (equal); methodology (equal); resources (equal); software (equal); writing – review and editing (equal). **Thanaporn Rungruang:** Conceptualization (equal); investigation (equal); methodology (lead); resources (lead); supervision (lead); validation (lead); writing – review and editing (supporting). **Narawut Pakaprot:** Conceptualization (equal); investigation (equal); methodology (equal); resources (lead); supervision (equal); writing – review and editing (supporting). **Pinidphon Prombutara:** Data curation (equal); formal analysis (equal); investigation (equal); resources (equal); software (equal); supervision (supporting); visualization (supporting); writing – review and editing (supporting). **Sujira Mukda:** Conceptualization (equal); investigation (equal); methodology (lead); resources (equal); supervision (lead); writing – review and editing (supporting). **Aurawan Kringkasemsee Kettawan:** Formal analysis (equal); resources (equal). **Aikkarach Kettawan:** Conceptualization (lead); formal analysis (equal); funding acquisition (lead); investigation (equal); project administration (lead); resources (lead); supervision (lead); writing – review and editing (equal).

## FUNDING INFORMATION

This research and innovation activity is funded by National Research Council of Thailand (NRCT), Grant number: N41D640036.

## CONFLICT OF INTEREST STATEMENT

There are no conflicts of interest.

## Supporting information


Data S1.

## Data Availability

The data that support the findings of this study are available from the corresponding author upon reasonable request.
