# Supplementary material for: Cold‐pressed perilla seed oil: Investigating its protective influence on the gut–brain axis in mice with rotenone‐induced Parkinson's disease
Source: Food Sci Nutr. 2024 Jun 14;12(9):6259–83. doi: 10.1002/fsn3.4265 (PMC11561828; doi:10.1002/fsn3.4265)
Supplement: Supplementary file 1 — Data S1. [file FSN3-12-6259-s001.docx]

# **Supplementary Materials**


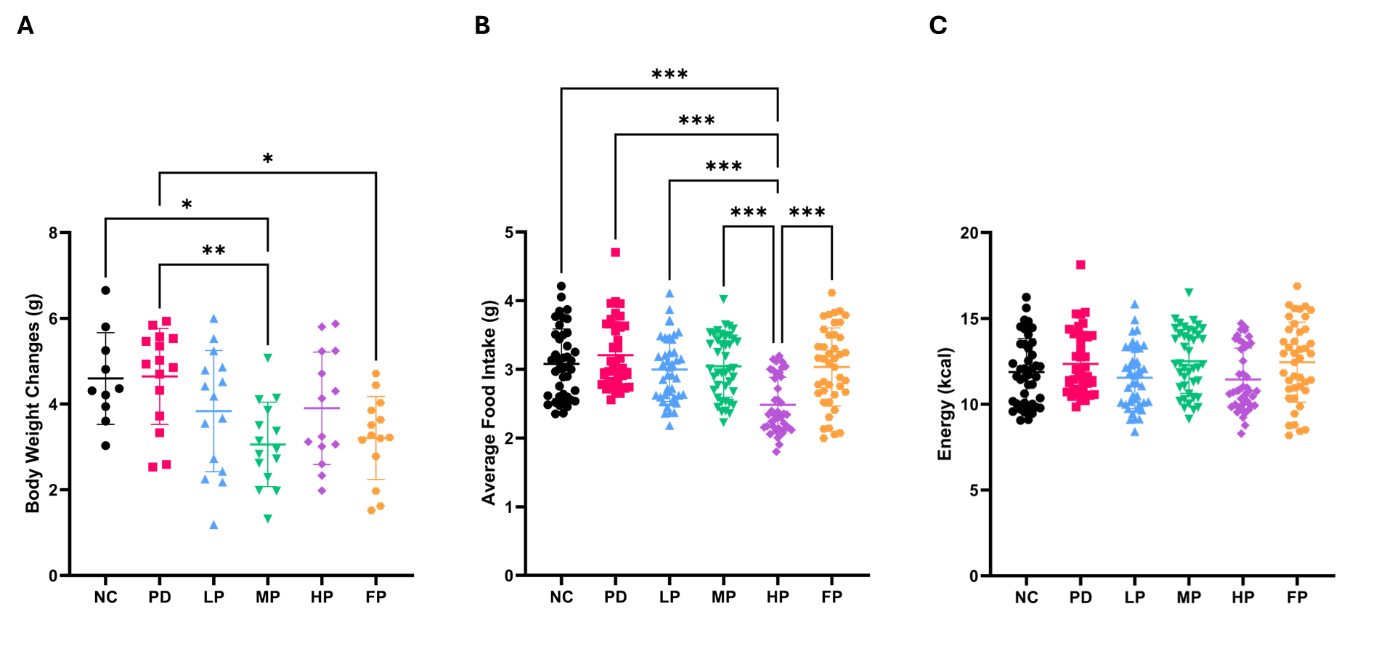


**Supplementary Figure S1** Changes in body weight (A) are shown as the difference between the initial and final body weight of mice in each group. The average daily food intake per mouse (B) was recorded over 42 days. The energy intake per mouse (C) was calculated based on the energy content of each experimental diet. The data, expressed as mean ± S.D., were analyzed using one-way ANOVA followed by Tukey’s multiple comparisons. Asterisks indicate significant differences (^*^ P < 0.05, ^**^ P < 0.01, ^***^ P < 0.001).

**Supplementary Table S1** Animal diet

| **AIN-93M diet formulated for maintenance of adult rodents** | | | | | | |
| --- | --- | --- | --- | --- | --- | --- |
| **Ingredients (g/kg)** | **NC** | **PD** | **LP** | **MP** | **HP** | **FP** |
| Cornstarch | 455.69 | 455.69 | 455.69 | 405.69 | 305.69 | 405.69 |
| Casein | 140.00 | 140.00 | 140.00 | 140.00 | 140.00 | 140.00 |
| Dextrinized cornstarch | 155.00 | 155.00 | 155.00 | 155.00 | 155.00 | 155.00 |
| Sucrose | 100.00 | 100.00 | 100.00 | 100.00 | 100.00 | 100.00 |
| Soybean oil | 50.00 | 50.00 |  |  |  |  |
| Fish oil |  |  |  |  |  | 100.00 |
| Perilla seed-oil |  |  | 50.00 | 100.00 | 200.00 |  |
| Fiber | 50.00 | 50.00 | 50.00 | 50.00 | 50.00 | 50.00 |
| Mineral mix | 35.00 | 35.00 | 35.00 | 35.00 | 35.00 | 35.00 |
| Vitamin mix | 10.00 | 10.00 | 10.00 | 10.00 | 10.00 | 10.00 |
| L-Cystine | 1.80 | 1.80 | 1.80 | 1.80 | 1.80 | 1.80 |
| Choline bitartrate | 2.50 | 2.50 | 2.50 | 2.50 | 2.50 | 2.50 |
| Tert-butylhydroquinone | 0.01 | 0.01 | 0.01 | 0.01 | 0.01 | 0.01 |
| Total | 1000.00 | 1000.00 | 1000.00 | 1000.00 | 1000.00 | 1000.00 |
| Total energy | 3852.77 | 3852.77 | 3852.77 | 4102.77 | 4602.77 | 4102.77 |
| %Energy carbohydrate | 73.79 | 73.79 | 73.79 | 64.41 | 48.73 | 64.41 |
| %Energy protein | 14.54 | 14.54 | 14.54 | 13.65 | 12.17 | 13.65 |
| %Energy fat | 11.68 | 11.68 | 11.68 | 21.94 | 39.11 | 21.94 |
| %Total | 100.00 | 100.00 | 100.00 | 100.00 | 100.00 | 100.00 |

**Supplementary Table S2** Estimated average energy from fat, fat consumption, and n-3 PUFA intake per mouse in each study group, calculated based on the experimental diet formulas.

|  | **Energy from fat (kcal)** | **Fat intake (g)** | **n-3 PUFA (g)** |
| --- | --- | --- | --- |
| **NC** | 1.386 ± 0.23 | 0.1541 ± 0.03 | 0.0077 ± 0.0013 |
| **PD** | 1.443 ± 0.22 | 0.1603 ± 0.02 | 0.0080 ± 0.0012 |
| **LP** | 1.349 ± 0.21 | 0.1499 ± 0.02 | 0.0920 ± 0.0143 ^a,b^ |
| **MP** | 2.742 ± 0.41 ^a,b,c^ | 0.3047 ± 0.05 ^a,b,c^ | 0.1869 ± 0.0279 ^a,b,c,e^ |
| **HP** | 4.472 ± 0.72 ^a,b,c,d^ | 0.4969 ± 0.08 ^a,b,c,d^ | 0.3049 ± 0.0491 ^a,b,c,d^ |
| **FP** | 2.732 ± 0.51 ^a,b,c,e^ | 0.3035 ± 0.06 ^a,b,c,e^ | 0.0786 ± 0.0147 ^a,b,c,d,e^ |

The n-3 PUFA content in commercially available soybean oil, menhaden fish oil (Sigma-Aldrich, CAS no. 8002-50-4, batch no. SLCD8682), and perilla seed oil were 5%, 25.9%, and 61.36%, respectively. Superscript letters indicate significant differences compared to: ^a^, NC; ^b^, PD; ^c^, LP; ^d^, MP; and ^e^, HP (P < 0.001 for all comparisons).
